# Choosing Important Health Outcomes for Comparative Effectiveness Research: A Systematic Review

**DOI:** 10.1371/journal.pone.0099111

**Published:** 2014-06-16

**Authors:** Elizabeth Gargon, Binu Gurung, Nancy Medley, Doug G. Altman, Jane M. Blazeby, Mike Clarke, Paula R. Williamson

**Affiliations:** 1 University of Liverpool, Department of Biostatistics, Liverpool, United Kingdom; 2 University of Oxford, Centre for Statistics in Medicine, Botnar Research Centre, Oxford, United Kingdom; 3 School of Social and Community Medicine, University of Bristol, Bristol, United Kingdom; 4 Queens University Belfast, Institute of Clinical Sciences, Block B, Royal Hospitals, Belfast, United Kingdom; Johns Hopkins Bloomberg School of Public Health, United States of America

## Abstract

**Background:**

A core outcome set (COS) is a standardised set of outcomes which should be measured and reported, as a minimum, in all effectiveness trials for a specific health area. This will allow results of studies to be compared, contrasted and combined as appropriate, as well as ensuring that all trials contribute usable information. The COMET (Core Outcome Measures for Effectiveness Trials) Initiative aims to support the development, reporting and adoption of COS. Central to this is a publically accessible online resource, populated with all available COS. The aim of the review we report here was to identify studies that sought to determine which outcomes or domains to measure in all clinical trials in a specific condition and to describe the methodological techniques used in these studies.

**Methods:**

We developed a multi-faceted search strategy for electronic databases (MEDLINE, SCOPUS, and Cochrane Methodology Register). We included studies that sought to determine which outcomes/domains to measure in all clinical trials in a specific condition.

**Results:**

A total of 250 reports relating to 198 studies were judged eligible for inclusion in the review. Studies covered various areas of health, most commonly cancer, rheumatology, neurology, heart and circulation, and dentistry and oral health. A variety of methods have been used to develop COS, including semi-structured discussion, unstructured group discussion, the Delphi Technique, Consensus Development Conference, surveys and Nominal Group Technique. The most common groups involved were clinical experts and non-clinical research experts. Thirty-one (16%) studies reported that the public had been involved in the process. The geographic locations of participants were predominantly North America (n = 164; 83%) and Europe (n = 150; 76%).

**Conclusions:**

This systematic review identified many health areas where a COS has been developed, but also highlights important gaps. It is a further step towards a comprehensive, up-to-date database of COS. In addition, it shows the need for methodological guidance, including how to engage key stakeholder groups, particularly members of the public.

## Introduction

Clinical trials seek to evaluate whether interventions are effective and safe for patients by comparing their relative effects on outcomes chosen to identify benefits and harms. Decision makers can then use this information to make well-informed healthcare choices. Therefore, it is critical that the outcomes measured and reported in trials are those that are needed by decision makers. However, inadequate attention to the choice of outcomes in clinical trials has led to avoidable waste in the production and reporting of research, and the outcomes included in research have not always been those that patients regard as most important or relevant [1].

It has been widely shown that inconsistencies in outcomes cause problems for people trying to use healthcare research. One such example was a recently published cross-sectional study of oncology research that found that more than 25,000 outcomes had appeared only once or twice in oncology trials [2]. Furthermore, key outcomes may go unreported, and a review of missing data in Cochrane Reviews found that 102/143 (71%) reviews were unable to obtain the findings for key outcomes in the included trials, and 26 (18%) were missing data for more than half the patients on the review's pre-specified primary outcome [3]. There are also often differences in how outcomes are defined and measured making it difficult, or impossible, to synthesise the results of different research studies and apply them in a meaningful way. For example, a recent survey of trials involving people with schizophrenia found that 2194 different scales had been used in 10,000 controlled trials: on average, a new instrument had been introduced for every fifth trial [4].

Alongside inconsistency in the measurement of outcomes, outcome reporting bias adds to the problems faced by users of research. This occurs if the results of an analysis are used to choose which outcomes will be reported. This causes bias, because the selectively un-reported results would remain un-accessible to users of the research [5]. These inconsistencies and bias in the availability of data on the effects of interventions could be addressed with the development and application of agreed standardised sets of outcomes, known as core outcome sets (COS), that should be measured and reported as a minimum in all effectiveness trials for a specific health area [6]. The COMET (Core Outcome Measures in Effectiveness Trials) Initiative (www.comet-initiative.org) brings together people interested in the development, reporting and application of COS. These sets are also suitable for use in clinical audit or research other than randomised trials. The existence or use of a COS does not imply that outcomes in a particular trial should be restricted to those in the relevant set. Rather, the expectation is that the core outcomes will always be collected and reported ***as a minimum***, making it easier for the results of trials to be compared, contrasted and combined as appropriate, while researchers might also include other outcomes of particular relevance to their specific study. COMET aims to collate and stimulate relevant resources, both applied and methodological, to facilitate exchange of ideas and information, and to foster methodological research in the area of COS; by bringing all relevant material together and making it accessible.

For COS to be an effective solution, they need to be easily accessible to researchers and other key groups. They are currently scattered across the health literature, so we have set out to bring these resources together in one place, developing a unique inventory. We have developed a publically accessible internet-based resource to collate the knowledge base for COS development, as well as the applied work that has been done according to health area. This will include planned and ongoing work as well as published accounts of COS development. It builds on a review of studies that addressed which outcomes to measure in clinical trials in children, (conducted in 2006) which identified work in 17 different paediatric conditions [7]. This, and studies that had been identified in ad hoc ways, was the starting point for the COMET database. However, in order for the database to be comprehensive and up-to-date, a systematic approach is needed to identify relevant material. We designed the systematic review that we report here to identify studies which sought to determine which outcomes or domains to measure in all clinical trials in a specific condition, and to identify and describe the methodological techniques used in these studies.

## Methods

The protocol is available at http://www.comet-initiative.org/about/researchprojects.

### Study selection

#### Inclusion and exclusion criteria

We chose studies as eligible for inclusion if they had developed or applied methodology for determining which outcome domains or outcomes should be measured, or are important to measure, in clinical trials or other forms of health research. We categorised studies as ineligible if, instead, they were related to how, rather than which, outcomes should be measured; reported the design or rationale for a single trial; were related to preclinical or early phase trials only; reported the use of a COS*; were a systematic review of clinical trials; were studies or systematic reviews of studies of prognosis; were studies (including systematic reviews and surveys) of outcomes measured in clinical trials** or quantitative descriptions (e.g. frequency) of outcomes**; were based on the opinion of a single author only**or focussed on one domain/outcome only**.

* reports relating to COS but not meeting inclusion criteria (e.g. where a COS has been used) were retrieved, and their references checked for potentially eligible studies.

** although these were not included in the systematic review, they are eligible for inclusion in the COMET database.

#### Types of participants and interventions

We categorised studies as eligible if they related to participants of any age, with any health condition in any setting and assessing the effect of any intervention.

### Identification of relevant studies

In August 2013, we searched MEDLINE via Ovid, SCOPUS (including EMBASE) and Cochrane Methodology Register without date and language restrictions. We developed a multi-faceted search strategy using a combination of text words and index terms, adapting the search strategy as appropriate for each database. For full details of the search strategy see Table S1.

In addition to this database searching, we completed a range of hand searching activities, in keeping with research evidence showing the benefits of adding hand searching to electronic searching [8]. We identified and reviewed funded projects that included the development of a COS, including National Institute for Health Research (NIHR) programme grant scheme reports and Health Technology Assessment (HTA) reports; searched for known key authors and citations to key papers, for example, the work of the OMERACT (Outcome Measures in Rheumatology) group; examined references cited in eligible studies and in ineligible studies that referred to or used a COS.

We contacted the 50 Cochrane Review Groups (CRG) as of 2011 across all areas of health care to request information on COS that they were aware of (by asking “Are you aware of any other work already done/being done attempting to develop a core outcome set for conditions covered by your CRG?”). Full details of the methods used for that study can be found in Kirkham et al 2013 [3].

### Selecting studies for inclusion in the review

We combined the records from each database and removed duplicates. We read titles and abstracts to assess eligibility (stage 1) and obtained the full texts of potentially relevant articles to assess for inclusion (stage 2).

One reviewer (EG) read the title and abstract of each citation and independent checks were performed by a second reviewer (BG). If agreement could not be achieved, the citation was retained for future checking. One of three reviewers (EG, BG, or NM) assessed each full paper. If we judged an article to be ineligible at this stage, we documented the reason for exclusion.

### Checking for agreement between reviewers

We checked for agreement between reviewers at each stage of the review process. Reviewers independently assessed batches of abstracts (EG and BG) and full papers (EG, BG and NM) to check for agreement before independently assessing records.

### Checking for correct exclusion

We obtained full papers for a 1% sample of the records that had been excluded on the basis of the title and abstract and these were checked for correct exclusion by a second reviewer (NM). If any studies were found to have been excluded incorrectly, additional checking was performed within the other excluded records. We also assessed a minimum of 5% of the papers that were excluded after reading their full text, to check for correct exclusion at that stage.

### Data collection and extraction

A COS may be developed to cover all aspects of a disease or health condition, but it may also have been developed with a focus on a particular type of treatment only, or for a specific age group or stage of disease. It is therefore important in reporting the scope of a COS to consider the specific area of health or healthcare to which it applies, along with details of health condition, population (here we have focussed on age) and types of interventions [6]. We therefore extracted the following data as free text unless otherwise stated:

Study Details, including year of publication, study aims and intended use of COS recommendations; Health Area including disease or health category e.g. ‘Lungs & airways’ or ‘Pregnancy & childbirth’ (using a *checklist*) and disease name (e.g. ‘Asthma’); Target Population including age and type of intervention; Method of Development used; and Stakeholder Groups involved in the process (e.g. health professionals, public, industry) including geographical setting of participants. When using the term ‘public’ through this report we include patients, carers, health and social care service users and people from organisations who represent these groups [9].

### Data analysis and presentation of results

We report the review in accordance with PRISMA guidelines (see Checklist S1) [10].We describe the studies narratively, and present the findings in text and tables. We did not anticipate conducting any statistical analyses to combine the findings.

## Results

### Description of studies

**Figure 1:**
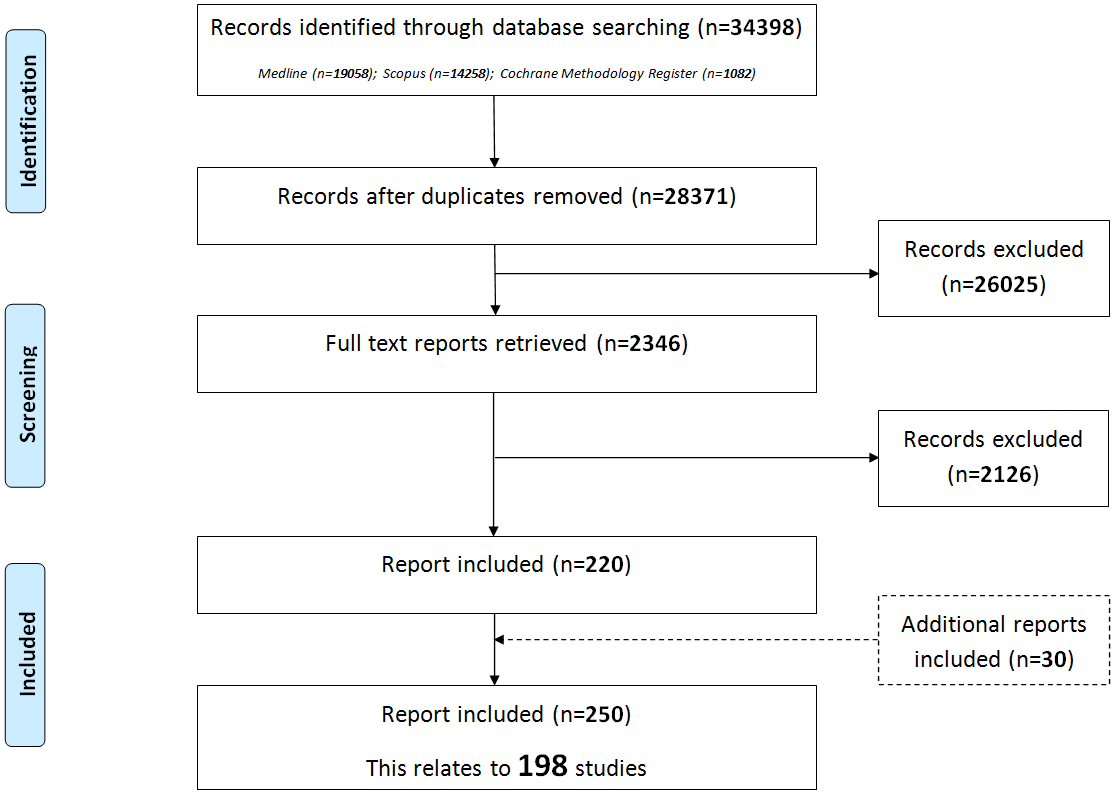
Identification of studies.

The initial database search identified 28,371 citations after duplicates had been removed. We excluded 26,025 records at the title and abstract stage, and 2126 after checking the full paper (Figure 1). A summary of the reasons for exclusion of the full papers is presented in Table 1. Two-hundred and twenty citations met the inclusion criteria. In addition to the database search, we identified 30 additional citations as eligible following reference checking. We did not identify any additional studies through the survey of Cochrane Review Groups. In total, we included 250 reports relating to 198 studies in the review (Table S2).

**Table 1:** Reasons for exclusion at stage 2 (assessment of full text reports).

Reason	n
Review/overview/discussion only, no outcome recommendations	495
Core outcomes/outcome recommendations not made	214
HRQL*1	117
How to measure outcome (including instruments, tools, scales, scores, outcome definition)	123
ICF core set development*2	80
Quality indicators – included an aspect of outcomes*3	78
Not relevant	669*5
ICF core set validation	56
Quality indicators – structure and/or process of care only	52
One outcome/domain only	40
Clinical management in practice not research (including for diagnosis)	45
Instrument development	24
Recommendation by single author only	21
Registry development*4	21
Describes features of registry	16
Preclinical/Early phase only (0, I, II)	18
On-going work	11
Duplicate	11
Quantitative description (e.g. frequency of symptoms)	9
Reporting the design/rationale of a single trial	22
Oral presentation only	2
Value attributed to outcomes	2
**TOTAL**	**2126**

*Although these studies are relevant to the development of a core outcome set (and therefore suitable for inclusion in the COMET database), they did not meet the review inclusion criteria.

*^1^These studies included qualitative studies describing the impact of a treatment or condition on a patient's quality of life, studies to determine particular domains of quality of life, and single patient narratives of the impact of a condition or treatment on their quality of life. The focus of these studies was on quality of life only.

*^2^Although the ICF is widely comprehensive [*13*] *, it is not all inclusive. For example, the ICF does not include outcomes such as death, an outcome that is often relevant to measure in clinical trials. Furthermore, as the ICF focuses on the individual only, caregiver outcomes would not be included. While for many health areas this may not be relevant, for some (e.g. dementia), caregiver outcomes may be core to measure.*

*^3^These studies assessed quality or efficiency of care (clinical practice), or the performance of an individual institution. Indicators were often specific to that scenario/environment of care only.

*^4^These studies described the development of registries, each with its own purpose, often to evaluate management of patients, identify best practices or to describe therapeutic strategies.

*^5^599 of these (90%) had no abstract to assess (title only), so had to be reviewed at full paper due to potential eligibility based on the title alone.

### Included studies

#### Year of publication

**Figure 2:**
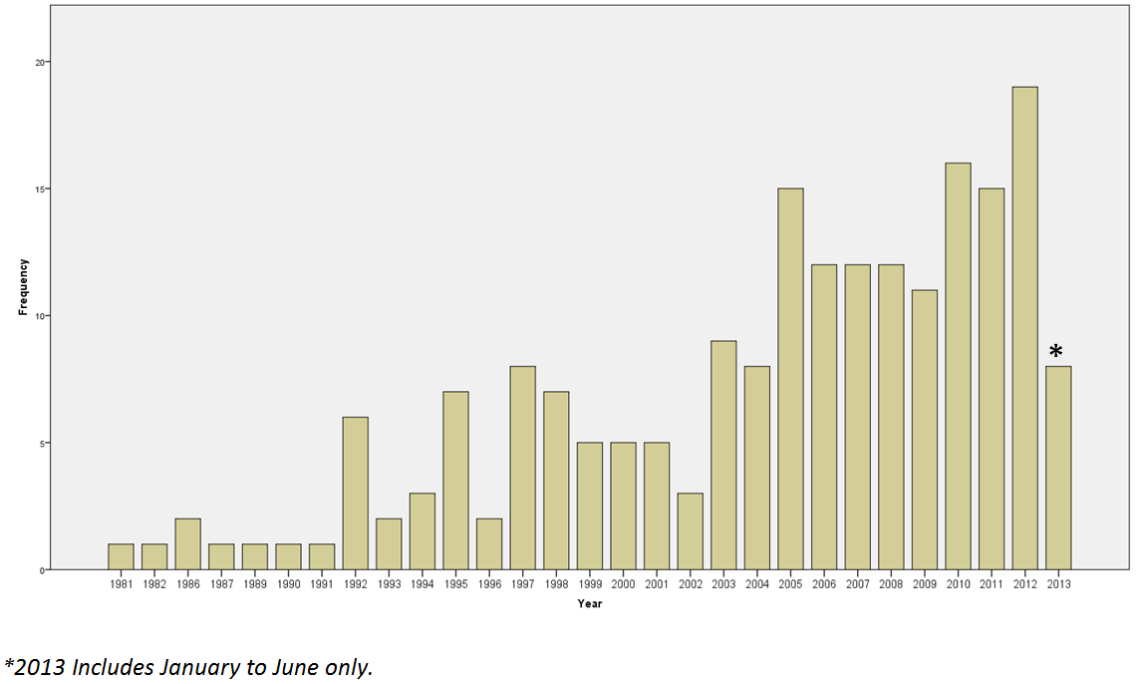
Year of first publication of each study (N = 198).

The year of publication of the earliest identified report for each study is shown in Figure 2, which clearly shows a general increase in the number of COS over the years.

#### Scope of core outcome sets

The scope of included studies is summarised in Table 2. This includes study aims, intended use, disease categories (classification according to disease name can be found in Table S2), population characteristics and intervention characteristics.

**Table 2:** The Scope of included studies (N = 198).

	n (%)
**Study aims**	
Considered outcomes while addressing wider clinical trial design issues (e.g. trial duration, ethical issues, eligibility criteria etc.)	101 (51)
Specifically addressed outcome selection and measurement	97 (49)
**Intended use of recommendations**	
Clinical trials	141 (71)
Clinical research	27 (14)
Clinical research and practice	11(6)
clinical trials and clinical practice	10 (5)
Clinical trials and regulatory purposes	3 (2)
Trials and observational studies	3 (2)
Observational studies	1 (<1)
Trials and case series	1 (<1)
Clinical research, clinical practice and regulatory purpose	1 (<1)
**Disease categories**	
Cancer	31 (16)
Rheumatology	28 (14)
Neurology	24 (12)
Heart & circulation	22 (11)
Dentistry & oral health	12 (6)
Infectious disease	12 (6)
Orthopaedics & trauma	10 (5)
Lungs & airways	8 (4)
Gastroenterology	8 (4)
Gynaecology	6 (3)
Tobacco, drugs, & alcohol dependence	4 (2)
Urology	4 (2)
Blood disorder	3 (2)
Anaesthesia & pain control	3 (2)
Mental health	3 (2)
Neonatal care	3 (2)
Skin	3 (2)
Others (chronic conditions, benign disease, intensive care)	3 (2)
Kidney disease	3 (2)
Pregnancy & childbirth	2 (1)
Endocrine & metabolic	2 (1)
Ear, Nose & Throat	1 (<1)
Genetic disorders	1 (<1)
Wounds	1 (<1)
Health care of older people	1 (<1)
**Population characteristics**	
All (adults and children stated explicitly)	13 (7)
Children	23 (12)
Adults	10 (5)
Older adults	3 (2)
Not specified	149 (75)
**Intervention characteristics**	
All intervention types	7 (4)
Drug treatments	40 (20)
*Drug only*	*34*
*Drug and rehabilitation*	*1*
*Drug and delivery management*	*1*
*Drug and physical therapy*	*1*
*Drug and complementary and alternative medicine (CAM) treatment*	*1*
*Immunomodulatory therapies*	*2*
Vaccine	2 (1)
Surgery	13 (7)
Procedure*	5 (3)
Device**	3 (2)
Other***	13 (7)
Not specified	115 (58)

*Procedure descriptions –.

*Procedure - Uterine artery embolization*.

*Procedure - Aortic valve stenosis (AS) - transcatheter aortic valve implantation*.

*Procedure - Aortic valve stenosis (AS)*.

*Procedure - pulp treatments of primary teeth*.

*Procedure - drug-eluting coronary stents (DES)*.

**Device descriptions –.

*Device – Compression (n = 2)*.

*Device - Mechanical circulatory support (MCS)*.

***Other descriptions –.

*Coronary angiogenesis*.

*Hip protectors*.

*Neuro-protective therapy (aka Neuroprotection)*.

*Non-surgical treatment (no other detail given)*.

*Operative and non-operative management*.

*Oral care products*.

*Ascorbic acid*.

*Exercise/physical activity*.

*Fall injury prevention interventions*.

*Behavioural therapies or other kinds of nonpharmacologic therapies*.

*Psychological & behavioural: Psychosocial*.

*Rehabilitation (vocational)*.

*Maternity care*.

#### Methods used to select outcomes

Studies reported using a variety of methods, sometimes in combination, to select the outcomes for the COS. The different methods used to select outcomes are shown in Table 3. The most frequent method used was semi-structured group discussion (n = 104, 54%), which included workshops (n = 39), meetings (n = 60), and round table discussion (n = 5). We classified a further 23 studies as using an unstructured group discussion (12%); descriptions included task forces, work(ing) groups/parties, committees, boards and panels. These studies did not describe whether they had face-to-face, telephone or electronic discussions. Sixty-five studies (33%) carried out a literature or systematic review. This was done in combination with another method in 54 of these 65 studies (83%). Other frequently used methods included the Delphi technique (n = 29, 15%), Consensus Development Conference (n = 20, 10%), Surveys (n = 17, 9%) and Nominal Group Technique (n = 15, 8%). More than one method was used in 74/198 (37%) studies. More detailed description about the combination of methods used can be found in Table 3. There was no description of the methods used in 16/198 (8%) studies.

**Table 3:** The methods used to develop core outcome sets (N = 198).

Main methods	n
**Semi-structured group discussion only**	**57**
*Workshop*	*22*
*Meeting (meeting, colloquium, conference where not described as consensus development conference)*	*32*
*Round table discussion*	*3*
**Unstructured group discussion only**	**18**
*Descriptions include task force, work group, working group/party, committee, board, panel*	
**Consensus development conference only**	**12**
**Literature/systematic review only**	**11**
**Delphi only**	**6**
**Survey only**	**3**
**NGT only**	**1**
**Mixed methods (** ***see descriptions below*** **)**	**74**
***Delphi + another method(s)***	***23***
*NGT*	*4*
*NGT + literature/systematic review*	*4*
*Semi-structured discussion (meeting& Workshop)*	*2*
*Systematic review + survey*	*1*
*Literature/systematic review*	*5*
*Literature/systematic review + semi-structured group discussion (meeting/workshop)*	*3*
*Literature/systematic review + meeting(s) + focus group(s) + workshop*	*1*
*Literature/systematic review + consensus conference*	*1*
*Literature/systematic review + survey + meeting*	*1*
*Meeting + survey*	*1*
***Semi-structured group discussion (listed which method) + another method(s)***	***29***
*Workshop + literature/systematic review*	*4*
*Meeting + literature/systematic review*	*13*
*Workshop and meeting*	*2*
*Workshop/meetings + web-based consultation*	*2*
*Workshop, literature/systematic review*	*1*
*Workshop + survey + literature/systematic review*	*3*
*Round table discussion + literature/systematic review*	*2*
*Meeting + focus group(s) + survey*	*1*
*Meeting + survey*	*1*
***Consensus development conference + another method(s)***	***7***
*Survey*	*1*
*NGT*	*1*
*Literature/systematic review*	*3*
*Meeting(s)*	*1*
*Literature/systematic review + survey + meeting*	*1*
***Unstructured group discussion + Literature/systematic review***	***5***
***NGT + another method(s)***	***5***
*Survey + interview*	*1*
*Semi-structured discussion (workshop & meetings)*	*1*
*Survey*	*1*
*Workshop + literature/systematic review*	*1*
*Literature review*	*1*
***Survey + Literature/systematic review***	***1***
***Focus group + rating exercise***	***1***
***Literature/systematic review, public presentation and debate***	***2***
***Literature/systematic review, survey and open discussion***	***1***
**No methods described**	**16**

#### People involved in selecting outcomes

Table 4 shows the participant groups that were included in these studies. Table 5 shows the participants' geographical location according to continent, as reported in the articles, as well as the median and range of number of countries included. In 34 studies, locations for participants other than the lead contact/participating authors were not provided. The geographic locations of participants were predominantly North America (n = 164; 83%) and Europe (n = 150; 76%). The remaining continents were represented in less than a quarter of studies; Australasia (n = 47; 24%), Asia (n = 40; 20%), South America (n = 23; 12%) and Africa (n = 13; 7%). The number of countries involved in the development of a COS ranged from 1 to 46 (a median of 4).

**Table 4:** Participant groups involved in selecting outcomes.

Participants category (total number of studies involving this particular participant category)	Sub-category (not mutually exclusive)	n (Frequency of the sub-category participants)
**Clinical experts (n = 173/198)**	Clinical experts*	88
	Clinical research expertise**	67
	Clinical trialists/Members of a clinical trial network	10
	Others with assumptions***	54
**Public representatives (n = 31/198)**	Patients	20
	Carers	7
	Patient support group representatives	9
	Service Users	2
**Non-clinical research experts (n = 54/198)**	Researchers	26
	Statisticians	20
	Epidemiologists	11
	Academic research representatives	4
	Methodologists	6
	Economists	3
**Authorities (n = 40/198)**	Regulatory agency representatives	31
	Governmental agencies	12
	Policy makers	4
	Charities	1
**Industry Representatives (n = 32/198)**	Pharmaceutical industry representatives	29
	Device manufacturers	2
	Biotechnology company representatives	1
**Others (n = 72/198)**	Ethicists	1
	Journal editors	2
	Others**** (besides known participants)	15
	Others with assumptions***	54
**No details given (n = 24/198)**		

* clinical experts includes multiple descriptions.

**16 studies, participants described as ‘researchers/investigators’ or ‘academic researchers’.

*** 54 studies with clinical input but unclear about involvement of other stakeholders.

**** Workshop/meeting participants (*5), subcommittee/committee (*2), guidelines panel, military personnel, moderator and audience, representatives from EORTC, members with expertise in information technologies, informatics, clinical registries, data-standards development, expertise in vaccine safety, malaria control and representatives from funding agencies/registration authorities, and donor organisation, members of the Rheumatology Section of the American Academy of Pediatrics, the Pediatric Section of the ACR, and the Arthritis Foundation, the diagnostic radiology and basic science communities, and from individuals conversant with functional and quality of life (QOL) assessments, comparative effectiveness research, and cost/benefit analysis.

**Table 5:** Participants' geographical location.

Continents	n (%)	Median and range of number of countries
North America and Europe^1^ *	56 (28)	4, 2–25
North America^2^	44 (22)	1, 1–2
Europe^3^	32 (16)	2, 1–14
North America, Europe and Australasia^4^ *	13 (7)	7, 3–25
North America, Europe and Asia^5^	11 (6)	9, 5–14
North America, Europe, Australasia, Asia^4^	10 (5)	11, 6–15
North America, South America, Europe, Australasia and Asia^4^ *	10 (5)	16, 5–21
North America, South America, Europe, Australasia, Asia and Africa*	4 (2)	26, 8–46
North America, Europe, Australasia and Africa^5^ **	3 (2)	8, 3–17
North America and Australasia	2 (1)	3, 3
North America, South America and Europe	2 (1)	10, 9–11
North America, South America, Europe and Asia	2 (1)	11, 7–15
Australasia	1 (<1)	2
North America, Europe and Africa	1 (<1)	10
North America, South America, Asia and Africa	1 (<1)	5
North America, South America, Europe and Australasia	1 (<1)	11
North America, South America, Europe and Africa	1 (<1)	7
North America, Europe, Australasia, Asia and Africa	1 (<1)	15
North America, South America, Europe, Australasia and Africa	1 (<1)	8
North America, South America, Europe, Asia and Africa	1 (<1)	18
Europe and Australasia	1 (<1)	2

*Besides the lead contact or participating authors, other participants' locations were not stated/known (^1^ – 15 studies, ^2^ - 9 studies, ^3^ - 7 studies, ^4^ - 2 studies, ^5^ - 1 study).*

* In 6 studies, OMERACT participants' information was extracted from the introductory paper.

** In 1 study, participants' location was based on where they had graduated from.

The types of people who are regarded as (or determined to be) key to developing a COS will vary between clinical areas, but two stakeholder groups that are likely to be important to all COS are clinical experts and the public. Where the types of people involved were described in the studies in this review, we found that almost all the COS included clinical experts (173/174 studies). We found that only 18% (31/174) included public representatives in this process. Public representatives were identified most commonly via medical institutions (n = 10), and four of these studies also used a charity or support group to identify public participants. However, the majority of studies that included public representatives did not describe how they were identified (18/31 studies, 58%). The number of public representatives that they included was not reported in 11 studies. A description of the methods used, the number of public representatives involved and the proportion of the total participants this represents is given in Table 6. It was not always clear what part of the COS development process they were involved in (12/31 studies, 39%). In 12 studies, they were involved in generating a list of outcomes and prioritisation of outcomes, and the remaining seven studies included public representatives in the prioritisation of outcomes stage only. Only three studies provided some description of how the material for explaining outcomes was developed for this group of stakeholders. In two studies, clinicians explained verbally what was meant. One of these studies, and an additional study, also carried out a pilot phase where public representatives were asked whether the questions or items were easy to understand and appropriate, and the wording was then refined accordingly.

**Table 6:** Public involvement detail (N = 20).

	Method	Total number of participants n	Number of public participants n	% public participants
1	Delphi (mixed panel) *- Number of rounds not clear, all took part in all rounds*	10	1	10%
2	Consensus Process (guidelines for trials) - review of RCTs and open discussion	6	2	33%
	Survey (mixed)	461	Not reported	Unknown *- Of 335 suggestions, 68% were from patients*
3*	Workshops (mixed)	OMERACT 6: 57	OMERACT 6: 11	OMERACT 6: 19%
		OMERACT 7: 179	OMERACT 7: 19	OMERACT 7: 11%
	Meeting (mixed)	OMERACT 8: 80	OMERACT 8: 20	OMERACT 8: 25%
4**	Interviews (patient only)	23	23	100%
	Nominal Group Technique (patient only)	26	26	100%
	Postal survey (patient only)	254	254	100%
5	Focus groups (mixed)	27	12	45%
	Rating exercise (mixed)	38	19	50%
6	Surveys (parents and children) and delphi (clinicians) - *same study*	Round 1: 95	Round 1: 49	Round 1: 52%
		Round 2: 93	Round 2: 50	Round 2: 54%
7	SR and survey (mixed)	12	6	50%
	Delphi (mixed)	46	6	13% (same for all 3 rounds)
	Meeting(mixed)	43	5	12%
8	Delphi (mixed)	Round 1: 83	Round 1: 44	Round 1: 53%
		Round 2: 75	Round 2: 38	Round 2: 51%
		Round 3: 68	Round 3: 32	Round 3: 47%
9*	Focus groups (patient only)	31	31	100%
	Survey(patient only)	959	959	100%
10	Focus groups (patient only)	48	48	100%
	Delphi (patient only)	Pretest: 100	Pretest: 100	100%
	*Did separate patient and researcher Delphi*	Round 1: 73	Round 1: 73	
		Round 2: 84	Round 2: 84	
	OMERACT 9 module (mixed)	not clear	not clear	Unknown
11	Rating exercise (mixed)	13	3	23%
12	Delphi (mixed)	Round 1: 218	Round 1: 9	Round 1: 4%
		Round 2: 173	Round 2: Not reported	Round 2: Unknown
		Round 3: 152	Round 3: 5	Round 3: 3%
13	Advisory panel meeting (mixed)	11	2	18%
14	Step 4 - survey and meeting (mixed)	Step 4–6	2	step 4 (33%)
	Step 6 - Delphi (mixed - round 3 only related to outcomes - previous rounds related to priority research questions)	Step 6–9		step 6 (22%)
15	Delphi (mixed) *- rounds not reported*	338	86	25%
16	Consensus conference (mixed)	36	2	6%
17	Survey (mixed)	136	5	4%
18	Workshop (mixed)	39	2	5%
19	Workshop (mixed)	23	1	4%
20	Workshop (mixed)	23	2	9%

*COS had already been developed without patient input, so this work done to elicit patient opinion.

** *Patient core set.*

## Discussion

This study provides the first complete assessment of COS that have been developed to standardise the outcomes being measured and reported in health research. We identified 198 studies, in a range of health areas, and demonstrate that there has been a rapid increase in the development of COS over recent years. The studies identified in this review have been included in the COMET database, which also includes planned and on-going COS development studies. As of December 2013, there are 51 reports of on-going studies in the COMET database, along with a further 40 potential areas of work that have been identified by particular research groups.

Although a wide range of health areas were identified in our review, we found that some are more active in this field than others. This review allows the identification of areas where COS may be lacking, and these gaps provide future opportunities for COS developers. Developers need to define the scope of the COS set at the outset in terms of health condition, population and types of interventions [6]. This review suggests that this has not always been done or is not described adequately in the reports, which also suggests a need for better reporting of studies of COS development.

A striking aspect of the results is the infrequency with which public representatives have been involved in the development of COS. Clinical trials are undertaken to establish whether interventions work and are safe for patients, so it is critical to include outcomes that they consider to be important. We found that only 16% of studies (31/198 studies) included public representatives in the development process, highlighting a need to find ways of engaging this group of stakeholders in particular in future projects, as well as other stakeholder groups who would be relevant to the COS. Most of the included studies included participants from more than one continent, but were dominated by North America and Europe. COS developers should consider including collaborators from other places as well; especially if a COS is to be applicable to, and adopted across, international settings.

### Strengths and limitations of the review

We developed the search strategy in an iterative and methodological way to be highly sensitive, so that as many potentially relevant studies as possible were retrieved. Although every attempt was made to capture all relevant studies, a consequence of the lack of consistent indexing could be that some relevant studies were missed, along with studies that have been reported in journals and other places that were not indexed in the databases we searched. We carried out hand-searching activities to try and minimise this. We searched in multiple databases, but these do have a bias towards research from North America and Europe. However, future efforts to identify COS and to minimise potential waste through unnecessary duplication would be for the bibliographic databases to introduce an indexing term to make them easier to find. Another limitation is that we were unable to undertake a formal quality assessment of the included studies. This is because defining the quality of a COS is not straight forward, and no validated way of doing this has been developed to date. There is an urgent need to develop such an instrument, not least to help users appraise the quality and relevance of a COS to their research and practice.

Finally, it is worth noting again that the first step in COS development is typically ‘what to measure’, which is the focus of this review; while the ‘how’ and ‘when’ usually come later. In this review we only included studies that addressed the first part of the process but, as an aside, of the 198 studies included in this review, 75 (38%) contained recommendations about how to measure the outcomes in the COS.

### Implications

This systematic review provides a reliable evidence base for an online resource (www.comet-initiative.org). This is a freely accessible, publically available, searchable database that shows what work has been done in a particular health area. It will help to avoid unnecessary duplication of efforts and reduce waste in the production and reporting of research. Studies identified through this extensive review, which were not already included in the COMET database, have been added and an annual search of the literature will take place to keep the database current. The ready availability of COS should make it easier for researchers to design new trials. For example, the SPIRIT (Standard Protocol Items: Recommendations for Interventional Trials) guidance for protocols of clinical trials [11], includes a statement encouraging trial investigators to ascertain whether a COS exists relevant to their trial, and if so, to include those outcomes in their trial. The findings from this systematic review will help trialists to do this. Furthermore, applicants to the NIHR HTA programme in the UK, the Health Research Board in Ireland and the charity Arthritis Research UK, are now encouraged to consider COS when seeking funding for new trials. The COMET database will provide a resource for this.

The implications of our research go beyond clinical trials; with the developers of 11% of the COS we identified noting that they intended their recommendations for clinical practice, as well as health research. Furthermore, the National Institute for Health and Care Excellence (NICE) in the UK develops guidelines to help health and social care professionals deliver the best possible care based on the available evidence and, since 2009, has used standard criteria (Grading of Recommendations Assessment, Development and Evaluation, GRADE) to assess the quality of the evidence by outcome, rather than by study. In addition to these methods, NICE now emphasises checking of the COMET database in their guideline development process. This highlights the importance of the results of this review for the improved delivery of healthcare.

### Future work

The credibility of a COS depends on both the use of sound methodology in its development and transparent reporting of these methods. In this review, we highlight the need to improve the standards of reporting, and we have plans to develop guidelines for reporting studies. This will build on the preliminary checklist [6] based mainly on discussions among the COMET Management Group. We will follow the strategy proposed in EQUATOR guidelines [12] involving five major phases: initial steps, pre-meeting activities, face-to-face consensus meeting, post-meeting activities and post-publication activities.

This systematic review shows that a range of methods have been used, in a variety of ways, to develop COS. There is currently no accepted gold standard, and we will undertake in-depth qualitative interviews with COS developers to explore the variation in methods, and whether it might be possible to determine which methods are better or more appropriate than others. Furthermore, work is needed to assess the implications of different methods for minimising bias and maximising efficiency in the development of COS, and for ensuring uptake. We plan to develop a quality assessment instrument for studies developing COS, which will need to use criteria that are valid and reliable so that COS developers and users can assess the quality of a COS, helping in the decision about whether a COS is good enough to be adopted and, in some cases, in choosing between COS.

### Conclusion

We have reviewed studies that have addressed the development of COS for measurement and reporting in clinical trials. This review has brought together the existing research in a single place, and has provided a basis for improving standards for ongoing and future work to develop core outcome sets. We have highlighted future areas of research, including the need for methodological guidance for COS development, better indexing, the development of a quality assessment instrument and the identification of effective methods for engaging key stakeholder groups, in particular public representatives. Finally, we have shown that it is not always possible to identify key features of the development of a COS from the published report, highlighting a need for better reporting of COS development studies. We are undertaking further work to inform future guidelines for developing and reporting COS.

## Supporting Information

Table S1 **Search strategy.**
(DOCX)

Table S2 **Studies included in the systematic review (250 reports relating to 198 studies).**
(DOCX)

Checklist S1 **PRISMA checklist for content of a systematic review.**
(DOCX)
