# Supplementary material for: Choosing Important Health Outcomes for Comparative Effectiveness Research: A Systematic Review
Source: PLoS One. 2014 Jun 16;9(6):e99111. doi: 10.1371/journal.pone.0099111 (PMC4059640; doi:10.1371/journal.pone.0099111)
Supplement: Table S1 — Search strategy. (DOCX) [file pone.0099111.s001.docx]

**Supplementary information**

**Table S1: Search strategy**

| **Search terms for MEDLINE** | | | | |
| --- | --- | --- | --- | --- |
| 1 | | Health Services/ut [Utilization] | | |
| 2 | | registries/ | | |
| 3 | | systematic review.mp. | | |
| 4 | | structured review.ti. | | |
| 5 | | evidence based medicine.ab. | | |
| 6 | | exp Clinical Trials as Topic/ | | |
| 7 | | clinical trial$.ab. | | |
| 8 | | randomised controlled trial$.ti,ab. | | |
| 9 | | randomised trial$.ti,ab. | | |
| 10 | | 1 or 2 or 3 or 4 or 5 or 6 or 7 or 8 or 9 | | |
| 11 | | workgroup$.mp. | | |
| 12 | | standard$ outcome$.mp. | | |
| 13 | | Practice Guideline/ | | |
| 14 | | clinical database.mp. | | |
| 15 | | patient important outcome$.mp. | | |
| 16 | | (standard$ adj3 reporting).mp. | | |
| 17 | | congresses.pt. | | |
| 18 | | Delphi Technique/ | | |
| 19 | | (recommend$ adj3 outcome$).mp. | | |
| 20 | | consensus development conference.pt. | | |
| 21 | | outcome$ reporting.mp. | | |
| 22 | | priorit$ symptom$.mp. | | |
| 23 | | (task force adj3 outcome$).mp. | | |
| 24 | | appropriate outcome$.mp. | | |
| 25 | research design/ | | |  |
| 26 | | endpoint determination/ | | |
| 27 | | consensus development conference/ | | |
| 28 | | patient participation/ | | |
| 29 | | consensus.mp. | | |
| 30 | | workshop.mp. | | |
| 31 | | Consensus Development Conferences, NIH as Topic/ | | |
| 32 | | focus groups/ | | |
| 33 | | 11 or 12 or 13 or 14 or 15 or 16 or 17 or 18 or 19 or 20 or 21 or 22 or 23 or 24 or 25 or 26 or 27 or 28 or 29 or 30 or 31 or 32 | | |
| 34 | | outcome$.mp. | | |
| 35 | | end point$.mp. | | |
| 36 | | (core adj3 set).mp. | | |
| 37 | | treatment emergent problem$.mp. | | |
| 38 | | exp outcome Assessment Health Care/ | | |
| 39 | | Treatment Outcome/ | | |
| 40 | | Quality of Life/ | | |
| 41 | | 34 or 35 or 36 or 37 or 38 or 39 or 40 | | |
| 42 | | clinical-study design.mp. | | |
| 43 | | patient$ perspective$.ti. | | |
| 44 | | outcome$.mp. and delphi.ti. | | |
| 45 | | (outcome$ and delphi).ab. | | |
| 46 | | (perspective$ adj3 outcome$).ti. | | |
| 47 | | core outcome$.ti,ab. | | |
| 48 | | core set$.ti,ab. | | |
| 49 | | clinical trial design$.ti. | | |
| 50 | | design$ clinical trial$.ti. | | |
| 51 | | (consensus and outcome$).ti. | | |
| 52 | | 42 or 43 or 44 or 45 or 46 or 47 or 48 or 49 or 50 or 51 | | |
| 53 | | 10 and 33 and 41 | | |
| 54 | | 52 or 53 | | |
| **Search terms for SCOPUS** | | | | |
| ((((INDEXTERMS(registries)) OR (INDEXTERMS(clinical trials as topic)) OR (ABS("evidence based medicine")) OR (ABS("clinical trial*")) OR (INDEXTERMS("Health Services Utilization")) OR (TITLE-ABS-KEY("SYSTEMATIC REVIEW")) OR (TITLE("structured review"))) OR (TITLE OR ABS("randomised controlled trial*")) OR (TITLE OR ABS (randomised trial*))) AND (((TITLE-ABS-KEY(workgroup*)) OR (TITLE-ABS-KEY(standard* outcome*)) OR (INDEXTERMS(practice guideline)) OR (TITLE-ABS-KEY("clinical database")) OR (TITLE-ABS-KEY("patient important outcome*")) OR (TITLE-ABS-KEY("standard* outcome*")) OR (INDEXTERMS(delphi technique))) OR ((TITLE-ABS-KEY(recommend* W/3 outcome*)) OR (TITLE-ABS-KEY(standard* W/3 reporting*)) OR (TITLE-ABS-KEY(task force W/3 outcome*)) OR (TITLE-ABS-KEY("appropriate outcome*")) OR (TITLE-ABS-KEY("outcome* reporting")) OR (TITLE-ABS-KEY("priorit* symptom*")) OR (INDEXTERMS(focus group)) (INDEXTERMS(research design))) OR ((INDEXTERMS(endpoint determination)) OR (INDEXTERMS(consensus development conference)) OR (INDEXTERMS(patient participation)) OR (TITLE-ABS-KEY(consensus)) OR (TITLE-ABS-KEY(workshop)))) AND 74) OR (((TITLE("design* clinical trials")) OR (TITLE(consensus AND outcome*)) OR (TITLE-ABS-KEY("clinical-study design")) OR (TITLE("patient* perspective*")) OR (ABS(outcome* AND delphi)) OR (TITLE(outcome* AND delphi)) OR (TITLE(perspective* W/3 outcome*)) OR (ABS("core outcome*") OR TITLE("core outcome*"))) OR ((ABS("core set*") OR TITLE("core set*")) OR (TITLE("clinical trial design*")))) | | | | |
| **Search terms for Cochrane Methodology Register** | | | | |
| #1 | | | [(clinical trial*):ab](http://onlinelibrary.wiley.com.ezproxy.liv.ac.uk/o/cochrane/searchHistory?mode=runquery&qnum=1) | |
| #2 | | | [MeSH descriptor Health services](http://onlinelibrary.wiley.com.ezproxy.liv.ac.uk/o/cochrane/searchHistory?mode=runquery&qnum=2) | |
| #3 | | | [MeSH descriptor registries](http://onlinelibrary.wiley.com.ezproxy.liv.ac.uk/o/cochrane/searchHistory?mode=runquery&qnum=3) | |
| #4 | | | [(systematic review ):ti,ab,kw](http://onlinelibrary.wiley.com.ezproxy.liv.ac.uk/o/cochrane/searchHistory?mode=runquery&qnum=4) | |
| #5 | | | [(structured review ):ti](http://onlinelibrary.wiley.com.ezproxy.liv.ac.uk/o/cochrane/searchHistory?mode=runquery&qnum=5) | |
| #6 | | | [(evidence based medicine ):ab](http://onlinelibrary.wiley.com.ezproxy.liv.ac.uk/o/cochrane/searchHistory?mode=runquery&qnum=6) | |
| #7 | | | [MeSH descriptor Clinical Trials as Topic explode all trees](http://onlinelibrary.wiley.com.ezproxy.liv.ac.uk/o/cochrane/searchHistory?mode=runquery&qnum=7) | |
| #8 | | | [(randomised controlled trial):ti or (randomised controlled trial):ab](http://onlinelibrary.wiley.com.ezproxy.liv.ac.uk/o/cochrane/searchHistory?mode=runquery&qnum=8) | |
| #9 | | | [(randomised trial*):ti,ab,kw](http://onlinelibrary.wiley.com.ezproxy.liv.ac.uk/o/cochrane/searchHistory?mode=runquery&qnum=1) | |
| #10 | | | [(**#1** OR **#2** OR **#3** OR **#4** OR **#5** OR **#6** OR **#7** OR **#8 OR #9**)](http://onlinelibrary.wiley.com.ezproxy.liv.ac.uk/o/cochrane/searchHistory?mode=runquery&qnum=9) | |
| #11 | | | [(workgroup*):ti,ab,kw](http://onlinelibrary.wiley.com.ezproxy.liv.ac.uk/o/cochrane/searchHistory?mode=runquery&qnum=10) | |
| #12 | | | [MeSH descriptor Practice Guideline](http://onlinelibrary.wiley.com.ezproxy.liv.ac.uk/o/cochrane/searchHistory?mode=runquery&qnum=11) | |
| #13 | | | [(patient important outcome*):ti,ab,kw](http://onlinelibrary.wiley.com.ezproxy.liv.ac.uk/o/cochrane/searchHistory?mode=runquery&qnum=12) | |
| #14 | | | [(clinical database):ti,ab,kw](http://onlinelibrary.wiley.com.ezproxy.liv.ac.uk/o/cochrane/searchHistory?mode=runquery&qnum=13) | |
| #15 | | | [standard* NEAR/3 reporting](http://onlinelibrary.wiley.com.ezproxy.liv.ac.uk/o/cochrane/searchHistory?mode=runquery&qnum=14) | |
| #16 | | | [(congresses):pt](http://onlinelibrary.wiley.com.ezproxy.liv.ac.uk/o/cochrane/searchHistory?mode=runquery&qnum=15) | |
| #17 | | | [MeSH descriptor Delphi Technique explode all trees](http://onlinelibrary.wiley.com.ezproxy.liv.ac.uk/o/cochrane/searchHistory?mode=runquery&qnum=16) | |
| #18 | | | [recommend* NEAR/3 outcome](http://onlinelibrary.wiley.com.ezproxy.liv.ac.uk/o/cochrane/searchHistory?mode=runquery&qnum=17) | |
| #19 | | | [(consensus development conference):pt](http://onlinelibrary.wiley.com.ezproxy.liv.ac.uk/o/cochrane/searchHistory?mode=runquery&qnum=18) | |
| #20 | | | [(priorit* symptom*):ti,ab,kw](http://onlinelibrary.wiley.com.ezproxy.liv.ac.uk/o/cochrane/searchHistory?mode=runquery&qnum=19) | |
| #21 | | | [(task force NEAR/3 outcome*):ti,ab,kw](http://onlinelibrary.wiley.com.ezproxy.liv.ac.uk/o/cochrane/searchHistory?mode=runquery&qnum=20) | |
| #22 | | | [(appropriate outcome*):ti,ab,kw](http://onlinelibrary.wiley.com.ezproxy.liv.ac.uk/o/cochrane/searchHistory?mode=runquery&qnum=21) | |
| #23 | | | [MeSH descriptor Focus Groups explode all trees](http://onlinelibrary.wiley.com.ezproxy.liv.ac.uk/o/cochrane/searchHistory?mode=runquery&qnum=22) | |
| #24 | | | [MeSH descriptor Research Design](http://onlinelibrary.wiley.com.ezproxy.liv.ac.uk/o/cochrane/searchHistory?mode=runquery&qnum=23) | |
| #25 | | | [MeSH descriptor endpoint determination](http://onlinelibrary.wiley.com.ezproxy.liv.ac.uk/o/cochrane/searchHistory?mode=runquery&qnum=24) | |
| #26 | | | [MeSH descriptor consensus development conference](http://onlinelibrary.wiley.com.ezproxy.liv.ac.uk/o/cochrane/searchHistory?mode=runquery&qnum=25) | |
| #27 | | | [MeSH descriptor patient participation](http://onlinelibrary.wiley.com.ezproxy.liv.ac.uk/o/cochrane/searchHistory?mode=runquery&qnum=26) | |
| #28 | | | [(consensus):ti,ab,kw](http://onlinelibrary.wiley.com.ezproxy.liv.ac.uk/o/cochrane/searchHistory?mode=runquery&qnum=27) | |
| #29 | | | [(workshop):ti,ab,kw](http://onlinelibrary.wiley.com.ezproxy.liv.ac.uk/o/cochrane/searchHistory?mode=runquery&qnum=28) | |
| #30 | | | [(outcome*):ti,ab,kw](http://onlinelibrary.wiley.com.ezproxy.liv.ac.uk/o/cochrane/searchHistory?mode=runquery&qnum=29) | |
| #31 | | | [(end point*):ti,ab,kw](http://onlinelibrary.wiley.com.ezproxy.liv.ac.uk/o/cochrane/searchHistory?mode=runquery&qnum=30) | |
| #32 | | | [(core NEAR/3 set):ti,ab,kw](http://onlinelibrary.wiley.com.ezproxy.liv.ac.uk/o/cochrane/searchHistory?mode=runquery&qnum=31) | |
| #33 | | | [(treatment emergent problem*):ti,ab,kw](http://onlinelibrary.wiley.com.ezproxy.liv.ac.uk/o/cochrane/searchHistory?mode=runquery&qnum=32) | |
| #34 | | | [MeSH descriptor Outcome Assessment (Health Care) explode all trees](http://onlinelibrary.wiley.com.ezproxy.liv.ac.uk/o/cochrane/searchHistory?mode=runquery&qnum=33) | |
| #35 | | | [MeSH descriptor Treatment Outcome](http://onlinelibrary.wiley.com.ezproxy.liv.ac.uk/o/cochrane/searchHistory?mode=runquery&qnum=34) | |
| #36 | | | [MeSH descriptor quality of life](http://onlinelibrary.wiley.com.ezproxy.liv.ac.uk/o/cochrane/searchHistory?mode=runquery&qnum=35) | |
| #37 | | | [(**#30** OR **#31** OR **#32** OR **#33** OR **#34** OR **#35** OR **#36**)](http://onlinelibrary.wiley.com.ezproxy.liv.ac.uk/o/cochrane/searchHistory?mode=runquery&qnum=36) | |
| #38 | | | ["standard outcome*":ti,ab,kw](http://onlinelibrary.wiley.com.ezproxy.liv.ac.uk/o/cochrane/searchHistory?mode=runquery&qnum=37) | |
| #39 | | | ["outcome* reporting":ti,ab,kw](http://onlinelibrary.wiley.com.ezproxy.liv.ac.uk/o/cochrane/searchHistory?mode=runquery&qnum=38) | |
| #40 | | | [(**#11 OR #12** OR **#13** OR **#14** OR **#15** OR **#16** OR **#17** OR **#18** OR #19 OR **#20** OR **#21** OR **#22** OR **#23** OR **#24** OR **#25** OR **#26** OR **#27** OR **#28** OR #29 OR **#38** OR **#39**)](http://onlinelibrary.wiley.com.ezproxy.liv.ac.uk/o/cochrane/searchHistory?mode=runquery&qnum=39) | |
| #41 | | | [(#10 AND #37 AND #40)](http://onlinelibrary.wiley.com.ezproxy.liv.ac.uk/o/cochrane/searchHistory?mode=runquery&qnum=40) | |
| #42 | | | [(design* clinical trials):ti](http://onlinelibrary.wiley.com.ezproxy.liv.ac.uk/o/cochrane/searchHistory?mode=runquery&qnum=41) | |
| #43 | | | [(clinical-study design):ti,ab,kw](http://onlinelibrary.wiley.com.ezproxy.liv.ac.uk/o/cochrane/searchHistory?mode=runquery&qnum=42) | |
| #44 | | | [(patient* perspective*):ti](http://onlinelibrary.wiley.com.ezproxy.liv.ac.uk/o/cochrane/searchHistory?mode=runquery&qnum=43) | |
| #45 | | | [(outcome*):ti and (delphi):ti](http://onlinelibrary.wiley.com.ezproxy.liv.ac.uk/o/cochrane/searchHistory?mode=runquery&qnum=44) | |
| #46 | | | [(outcome*):ab and (delphi):ab](http://onlinelibrary.wiley.com.ezproxy.liv.ac.uk/o/cochrane/searchHistory?mode=runquery&qnum=45) | |
| #47 | | | [(perspective* NEAR/3 outcome*):ti](http://onlinelibrary.wiley.com.ezproxy.liv.ac.uk/o/cochrane/searchHistory?mode=runquery&qnum=46) | |
| #48 | | | [(core outcome*):ti or (core outcome*):ab](http://onlinelibrary.wiley.com.ezproxy.liv.ac.uk/o/cochrane/searchHistory?mode=runquery&qnum=47) | |
| #49 | | | [(core set):ti or (core set):ab](http://onlinelibrary.wiley.com.ezproxy.liv.ac.uk/o/cochrane/searchHistory?mode=runquery&qnum=48) | |
| #50 | | | [(clinical trial design*):ti](http://onlinelibrary.wiley.com.ezproxy.liv.ac.uk/o/cochrane/searchHistory?mode=runquery&qnum=49) | |
| #51 | | | [(outcome):ti and (consensus):ti](http://onlinelibrary.wiley.com.ezproxy.liv.ac.uk/o/cochrane/searchHistory?mode=runquery&qnum=50) | |
| #52 | | | [(#42 OR #43 OR #44 OR #45 OR #46 OR #47 OR #48 OR #49 OR #50 OR #51)](http://onlinelibrary.wiley.com.ezproxy.liv.ac.uk/o/cochrane/searchHistory?mode=runquery&qnum=51) | |
| #53 | | | [(#41 OR #52)](http://onlinelibrary.wiley.com.ezproxy.liv.ac.uk/o/cochrane/searchHistory?mode=runquery&qnum=52) | |
| #54 | | | [(#53)](http://onlinelibrary.wiley.com.ezproxy.liv.ac.uk/o/cochrane/searchHistory?mode=runquery&qnum=53) | |
