# Supplementary material for: Choosing Important Health Outcomes for Comparative Effectiveness Research: A Systematic Review
Source: PLoS One. 2014 Jun 16;9(6):e99111. doi: 10.1371/journal.pone.0099111 (PMC4059640; doi:10.1371/journal.pone.0099111)
Supplement: Table S2 — Studies included in the systematic review (250 reports relating to 198 studies). (DOCX) [file pone.0099111.s002.docx]

**Table S3: Studies included in the systematic review (250 reports relating to 198 studies)**

| **Study** | **Disease category** | **Disease name** |
| --- | --- | --- |
| Dixon 1987 [[1](#_ENREF_1)]** | Cancer | Hodgkin's disease and lymphoma |
| Glynne-Jones 2006 [[2](#_ENREF_2)]** | Cancer | Rectal cancer |
| Auvinen 1996 [[3](#_ENREF_3)]** | Cancer | Prostate cancer |
| Denis 1997 [[4](#_ENREF_4)]* | Cancer | Prostate cancer (early stage) |
| Scher 2004 [[5](#_ENREF_5)]* | Cancer | Prostate cancer (rising prostate-specific antigen) |
| Dawson 1998 [[6](#_ENREF_6)]* | Cancer | Prostate cancer |
| Middleton 1995 [[7](#_ENREF_7)]  Schellhammer 1997 [[8](#_ENREF_8)] ** | Cancer | Localized prostate cancer |
| Rajkumar 2011 [[9](#_ENREF_9)]* | Cancer | Myeloma |
| Chow on behalf of International Bone Metastases Consensus Working Party [[10](#_ENREF_10)]*  Chow 2002 [[11](#_ENREF_11)] | Cancer | Bone metastases |
| Partsch 2010 [[12](#_ENREF_12)]* | Cancer | Breast cancer related lymphedema (BCRL) |
| Hesketh 1998 [[13](#_ENREF_13)]*  Tonato 1998 [[14](#_ENREF_14)] | Cancer | Chemotherapy-induced nausea and vomiting |
| Mcvie 1992 [[15](#_ENREF_15)]* | Cancer | Chemotherapy-induced nausea and vomiting |
| Pallis 2011 [[16](#_ENREF_16)]** | Cancer | Solid tumours |
| Miller 1981 [[17](#_ENREF_17)]** | Cancer | Cancer (not specified) |
| Prorok 2010 [[18](#_ENREF_18)]* | Cancer | Cancer (not specified) |
| Punt [[19](#_ENREF_19)]** | Cancer | Colorectal cancer |
| Wils 1998 [[20](#_ENREF_20)]** | Cancer | Colorectal cancer (advanced) |
| Llovet 2008 [[21](#_ENREF_21)]* | Cancer | Hepatocellular carcinoma |
| Pagliusi 2004 [[22](#_ENREF_22)]** | Cancer | Human papillomavirus (Cervical cancer) |
| Lefebvre 2009 [[23](#_ENREF_23)]* | Cancer | Head and neck cancer |
| Adelstein 2012 [[24](#_ENREF_24)]* | Cancer | Head and neck cancer |
| Gridelli 2004 [[25](#_ENREF_25)]* | Cancer | Advanced non-small-cell lung cancer |
| Gridelli 2012 [[26](#_ENREF_26)]* | Cancer | Advanced non-small-cell lung cancer |
| Cheson 1999 [[27](#_ENREF_27)]**  Cheson 2007 [[28](#_ENREF_28)] | Cancer | Non-Hodgkin's lymphoma |
| Anderson 2008 [[29](#_ENREF_29)]** | Cancer | Leukaemia |
| Cheson 2003 [[30](#_ENREF_30)]* | Cancer | Acute Myeloid Leukaemia |
| Kulke 2011 [[31](#_ENREF_31)]* | Cancer | Neuroendocrine tumours |
| Bellm 2002 [[32](#_ENREF_32)]** | Cancer | Oral Mucositis (OM) |
| Du Bois 2005 [[33](#_ENREF_33)]*  Stuart 2011 [[34](#_ENREF_34)]  Thigpen 2011 [[35](#_ENREF_35)] | Cancer | Ovarian cancer |
| Comenzo 2012 [[36](#_ENREF_36)]* | Cancer | Systemic light-chain amyloidosis |
| Dorman 2009 [[37](#_ENREF_37)]* | Cancer | Dyspnoea or Breathlessness in Palliative Care |
| Renal Disease Subcommittee of the American College of Rheumatology Ad Hoc Committee on Systemic Lupus Erythematosus Response Criteria [[38](#_ENREF_38)]* | Rheumatology | Systemic lupus erythematosus |
| Bertsias 2009 [[39](#_ENREF_39)]*  Gordon 2009 [[40](#_ENREF_40)] | Rheumatology | Systemic lupus erythematosus |
| Smolen 1999 [[41](#_ENREF_41)]** | Rheumatology | Systemic lupus erythematosus |
| Ruperto 2003 [[42](#_ENREF_42)]** | Rheumatology | Juvenile systemic lupus erythematosus and juvenile dermatomyositis |
| White 1995 [[43](#_ENREF_43)]* | Rheumatology | Systemic sclerosis |
| Khanna 2008 [[44](#_ENREF_44)]** | Rheumatology | Systemic sclerosis |
| Clements 2012 [[45](#_ENREF_45)]* | Rheumatology | Systemic sclerosis - related arthritis |
| Khanna 2010 [[46](#_ENREF_46)]* | Rheumatology | Systemic sclerosis-associated interstitial lung disease |
| Merkel 2009 [[47](#_ENREF_47)]**  Merkel 2011 [[48](#_ENREF_48)] | Rheumatology | Small-vessel Vasculitis/ ANCA-associated Vasculitis |
| Hellmich 2007 [[49](#_ENREF_49)]* | Rheumatology | Systemic vasculitis, AAV, anti-neutrophil cytoplasmic antibody-associated vasculitis; Wegener’s granulomatosis |
| Felson 1993 [[50](#_ENREF_50)]** | Rheumatology | Rheumatoid arthritis |
| Fried 1993 [[51](#_ENREF_51)]**  Tugwell 1993 [[52](#_ENREF_52)]  Boers 1994 [[53](#_ENREF_53)]  Kirwan 2003 [[54](#_ENREF_54)]  Kirwan 2005 [[55](#_ENREF_55)]  Kirwan 2007 [[56](#_ENREF_56)] | Rheumatology | Rheumatoid arthritis |
| Sanderson 2010 [[57](#_ENREF_57)]**  Sanderson 2010 [[58](#_ENREF_58)] | Rheumatology | Rheumatoid arthritis |
| Bombardier 1982 [[59](#_ENREF_59)] ** | Rheumatology | Rheumatoid arthritis |
| Scott 1989 [[60](#_ENREF_60)]** | Rheumatology | Rheumatoid arthritis |
| Van Riel 1992 [[61](#_ENREF_61)]** | Rheumatology | Rheumatoid arthritis |
| Taylor 2005 [[62](#_ENREF_62)]**  Gladman 2005 [[63](#_ENREF_63)]  Gladman 2005 [[64](#_ENREF_64)]  Gladman 2007 [[65](#_ENREF_65)] | Rheumatology | Psoriatic arthritis |
| Miller 2001 [[66](#_ENREF_66)]** | Rheumatology | Idiopathic inflammatory myopathies (IIM) |
| Van Der Heijde 1997 [[67](#_ENREF_67)]** | Rheumatology | Ankylosing spondylitis |
| Wolfe 1999 [[68](#_ENREF_68)]** | Rheumatology | Rheumatic diseases |
| Mease 2005 [[69](#_ENREF_69)]**  Mease 2007 [[70](#_ENREF_70)]  Arnold 2008 [[71](#_ENREF_71)]  Mease 2008 [[72](#_ENREF_72)]  Carville 2008 [[73](#_ENREF_73)]  Choy 2009 [[74](#_ENREF_74)]  Mease 2009 [[75](#_ENREF_75)] | Rheumatology | Fibromyalgia syndrome |
| Salaffi 2012 [[76](#_ENREF_76)]** | Rheumatology | Fibromyalgia syndrome |
| Schumacher 2005 [[77](#_ENREF_77)]**  Schumacher 2007 [[78](#_ENREF_78)]  Taylor 2008 [[79](#_ENREF_79)]  Schumacher 2009 [[80](#_ENREF_80)] | Rheumatology | Gout |
| Giannini 1997 [[81](#_ENREF_81)]** | Rheumatology | Arthritis |
| Bellamy 1997 [[82](#_ENREF_82)]** | Rheumatology | Knee, hip and hand osteoarthritis |
| Heiligenhaus 2012 [[83](#_ENREF_83)]** | Rheumatology | idiopathic arthritis-associated uveitis (juvenile) |
| Bowman 2001 [[84](#_ENREF_84)]**  Pillemer 2005 [[85](#_ENREF_85)] | Rheumatology | Sjögren's syndrome |
| Cranney 1997 [[86](#_ENREF_86)]**  Guidelines for osteoporosis trials[[87](#_ENREF_87)] | Rheumatology | Osteoarthritis |
| Hammarlund 2012 [[88](#_ENREF_88)]** | Neurology | Parkinson's disease |
| Vellas 2008 [[89](#_ENREF_89)]** | Neurology | Alzheimer's disease |
| World Federation of Neurology Research Group 1995 [[90](#_ENREF_90)]*  Miller 1999 [[91](#_ENREF_91)] | Neurology | Amyotrophic lateral sclerosis/motor neurone disease |
| Leigh 2004 [[92](#_ENREF_92)]* | Neurology | Amyotrophic lateral sclerosis/motor neurone disease |
| Shankaran 2003 [[93](#_ENREF_93)]* | Neurology | Hypoxic-ischemic brain injury |
| Vargus-Adams 2009 [[94](#_ENREF_94)]** | Neurology | Cerebral palsy |
| Katona 2007 [[95](#_ENREF_95)]** | Neurology | Dementia |
| Moniz-Cook 2008 [[96](#_ENREF_96)]** | Neurology | Dementia |
| ILAE Commission 1998 [[97](#_ENREF_97)]* | Neurology | Epilepsy (newly diagnosed and chronic) |
| LaFrance 2006 [[98](#_ENREF_98)]* | Neurology | Seizures |
| Osborne 2001 [[99](#_ENREF_99)]**  Lux 2004 [[100](#_ENREF_100)] | Neurology | Infantile spasms  West syndrome (Epilepsy) |
| Mindell 2006 [[101](#_ENREF_101)]* | Neurology | Insomnia |
| Schumacher 2010 [[102](#_ENREF_102)]* | Neurology | Intracranial cerebral atherosclerosis |
| Whitaker 1995 [[103](#_ENREF_103)]** | Neurology | Multiple sclerosis |
| Chitnis 2012 [[104](#_ENREF_104)]*  Chitnis 2013 [[105](#_ENREF_105)] | Neurology | Multiple sclerosis |
| Penzien 2005 [[106](#_ENREF_106)]*  Penzien 2005 [[107](#_ENREF_107)]  Andrasik 2005 [[108](#_ENREF_108)] | Neurology | Headache |
| Tfelt-Hansen 1991[[109](#_ENREF_109)]*  Tfelt-Hansen 2000 [[110](#_ENREF_110)]  Tfelt-Hansen 2012 [[111](#_ENREF_111)] | Neurology | Migraine |
| Lipton 1995 [[112](#_ENREF_112)]* | Neurology | Cluster headache |
| Schoenen 1995 [[113](#_ENREF_113)]*  Bendtsen 2010 [[114](#_ENREF_114)] | Neurology | Tension-type headache |
| Hughes 2005 [[115](#_ENREF_115)]* | Neurology | Chronic Inflammatory Demyelinating Polyradiculoneuropathy and Multifocal  Motor Neuropathy |
| Merkies 2006 [[116](#_ENREF_116)]** | Neurology | Peripheral neuropathy |
| Reilly 2006 [[117](#_ENREF_117)]* | Neurology | Charcot-Marie-Tooth disease type 1A (CMT1A) |
| Clifton 1992 [[118](#_ENREF_118)]** | Neurology | Traumatic brain injury |
| Wilde 2010 [[119](#_ENREF_119)]** | Neurology | Traumatic brain injury |
| Duncan 2000 [[120](#_ENREF_120)]** | Heart & circulation | Acute stroke |
| Schellinger 2012 [[121](#_ENREF_121)]** | Heart & circulation | Acute stroke |
| Hoeper 2004 [[122](#_ENREF_122)]* | Heart & circulation | Pulmonary arterial hypertension |
| Distler 2008 [[123](#_ENREF_123)]** | Heart & circulation | Pulmonary arterial hypertension relates Systemic Sclerosis |
| Becker 2011 [[124](#_ENREF_124)]** | Heart & circulation | Cardiac arrest |
| Chiam 2008 [[125](#_ENREF_125)]** | Heart & circulation | Aortic valve stenosis (AS) |
| Leon 2011 [[126](#_ENREF_126)]**  Kappetein 2012 [[127](#_ENREF_127)] | Heart & circulation | Aortic stenosis (AS);  Valvular heart disease |
| Cutlip 2007 [[128](#_ENREF_128)]** | Heart & circulation | Obstructive coronary artery disease |
| Simons 2000 [[129](#_ENREF_129)]* | Heart & circulation | Ischemic heart disease |
| Conte 2009 [[130](#_ENREF_130)]** | Heart & circulation | Critical limb ischemia |
| Timaran 2011 [[131](#_ENREF_131)]* | Heart & circulation | Atherosclerosis |
| Stout 2012 [[132](#_ENREF_132)]* | Heart & circulation | Chronic leg edema |
| Nedeltchev 2010 [[133](#_ENREF_133)]** | Heart & circulation | Obstructive disease of supra-aortic arteries |
| Anderson 2013 [[134](#_ENREF_134)]* | Heart & circulation | Cardiovascular disease |
| Hausenloy 2013 [[135](#_ENREF_135)]* | Heart & circulation | Coronary heart disease |
| Labs 1999 [[136](#_ENREF_136)]* | Heart & circulation | Peripheral arterial occlusive disease (PAOD) |
| Mitchell 2011 [[137](#_ENREF_137)]** | Heart & circulation | Deep venous thrombosis and pulmonary embolism |
| Steg 2011 [[138](#_ENREF_138)]* | Heart & circulation | Acute coronary syndrome |
| O'Connell 2009 [[139](#_ENREF_139)]* | Heart & circulation | Acute heart failure syndromes (AKA Acute decompensated heart failure) |
| Higashida 2003 [[140](#_ENREF_140)]* | Heart & circulation | Acute  ischemic stroke |
| Calkins 2012 [[141](#_ENREF_141)]* | Heart & circulation | Atrial fibrillation |
| Kirchhof 2007 [[142](#_ENREF_142)]** | Heart & circulation | Atrial fibrillation |
| Buser 1997 [[143](#_ENREF_143)]* | Dentistry & oral health | Implants in regenerated bone |
| Page 1992 [[144](#_ENREF_144)]* | Dentistry & oral health | Periodontitis |
| Imrey 1994 [[145](#_ENREF_145)]* | Dentistry & oral health | Periodontitis |
| Lightfoot 2005 [[146](#_ENREF_146)]** | Dentistry & oral health | Chronic periodontitis (Anterior teeth) |
| Lightfoot 2005 [[147](#_ENREF_147)]** | Dentistry & oral health | Chronic periodontitis (Posterior teeth) |
| Weber 1997 [[148](#_ENREF_148)]* | Dentistry & oral health | Endentulous |
| Cochran 1998 [[149](#_ENREF_149)]* | Dentistry & oral health | Endentulous |
| Tonetti 2012 [[150](#_ENREF_150)]** | Dentistry & oral health | Endentulous |
| Smaïl-Faugerson 2013 [[151](#_ENREF_151)]** | Dentistry & oral health | Extensive tooth decay |
| Chilton 1986 [[152](#_ENREF_152)]* | Dentistry & oral health | Plaque and gingivitis |
| Council on Dental Therapeutics 1986 [[153](#_ENREF_153)]* | Dentistry & oral health | Supragingival dental plaque and gingivitis |
| Pitts 2004 [[154](#_ENREF_154)]* | Dentistry & oral health | Caries |
| Marshall 2005 [[155](#_ENREF_155)]** | Infectious disease | Sepsis and critical care |
| Goldstein 2005 [[156](#_ENREF_156)]* | Infectious disease | Sepsis and critical care |
| Wood 1995 [[157](#_ENREF_157)]* | Infectious disease | Herpes Zoster |
| Barlow 2003 [[158](#_ENREF_158)]** | Infectious disease | Community-acquired pneumonia |
| Spellberg 2008 [[159](#_ENREF_159)]* | Infectious disease | Community-acquired pneumonia |
| Powers 2010 [[160](#_ENREF_160)]*  Spellberg 2010 [[161](#_ENREF_161)] | Infectious disease | Hospital-acquired pneumonia and ventilator-associated pneumonia |
| McCracken 1992 [[162](#_ENREF_162)]* | Infectious disease | Acute bacterial meningitis |
| Alioum 2001 [[163](#_ENREF_163)]* | Infectious disease | HIV |
| Kirkby 2010 [[164](#_ENREF_164)]* | Infectious disease | Influenza |
| Nystrom 1990 [[165](#_ENREF_165)]* | Infectious disease | Intraabdominal infection |
| Cross 2005 [[166](#_ENREF_166)]** | Infectious disease | Leprosy |
| Moorthy 2007 [[167](#_ENREF_167)]**  Moorthy 2009 [[168](#_ENREF_168)] | Infectious disease | Malaria |
| Steeves 2007 [[169](#_ENREF_169)]** | Orthopaedics & trauma | Spinal cord injury |
| Bombardier 2000 [[170](#_ENREF_170)]** | Orthopaedics & trauma | Spinal disorders |
| Deyo 1998 [[171](#_ENREF_171)]** | Orthopaedics & trauma | Low back pain |
| Devogelaer 2003 [[172](#_ENREF_172)]* | Orthopaedics & trauma | Acute low back pain |
| Goldhahn 2008 [[173](#_ENREF_173)]** | Orthopaedics & trauma | Osteoporosis |
| Lynch 2013 [[174](#_ENREF_174)]** | Orthopaedics & trauma | ACL injury |
| Falder 2009 [[175](#_ENREF_175)]** | Orthopaedics & trauma | Burns |
| Reneman 2013 [[176](#_ENREF_176)]** | Orthopaedics & trauma | Muskuloskeletal pain (subacute and chronic) |
| Lamb 2005 [[177](#_ENREF_177)]** | Orthopaedics & trauma | Fall injury |
| Cameron 2010 [[178](#_ENREF_178)]* | Orthopaedics & trauma | Hip fracture |
| Smith 1996 [[179](#_ENREF_179)]** | Lungs & airways | Asthma |
| Reddel 2009 [[180](#_ENREF_180)]** | Lungs & airways | Asthma |
| Busse 2012 [[181](#_ENREF_181)]**  Fuhlbrigge 2012 [[182](#_ENREF_182)]  Akinbami 2012 [[183](#_ENREF_183)]  Szefler 2012 [[184](#_ENREF_184)]  Tepper 2012 [[185](#_ENREF_185)]  Cloutier 2012 [[186](#_ENREF_186)]  Krishnan 2012 [[187](#_ENREF_187)]  Wilson 2012 [[188](#_ENREF_188)] | Lungs & airways | Asthma |
| Sinha 2012 [[189](#_ENREF_189)]** | Lungs & airways | Asthma |
| Keim 2004 [[190](#_ENREF_190)]** | Lungs & airways | Respiratory distress |
| Canonica 2007 [[191](#_ENREF_191)]* | Lungs & airways | Respiratory allergy |
| Cazzola 2008 [[192](#_ENREF_192)]** | Lungs & airways | Chronic obstructive pulmonary disorder (COPD) |
| Task Group on Mucoactive Drugs 1994 [[193](#_ENREF_193)]* | Lungs & airways | Chronic bronchitis and COPD |
| Dent 2008 [[194](#_ENREF_194)]* | Gastroenterology | Gastro-oesophageal reflux disease (GERD) |
| Wirth 2011 [[195](#_ENREF_195)]* | Gastroenterology | Chronic Hepatitis C |
| Bajaj 2011 [[196](#_ENREF_196)]* | Gastroenterology | Hepatic encephalopathy |
| Fekety 1992 [[197](#_ENREF_197)]* | Gastroenterology | Antibiotic associated colitis |
| Griffiths 2005 [[198](#_ENREF_198)]** | Gastroenterology | Crohn's disease |
| Laine 2010 [[199](#_ENREF_199)]* | Gastroenterology | Nonvariceal upper gastrointestinal bleeding |
| Pimentel 2013 [[200](#_ENREF_200)]* | Gastroenterology | Irritable bowel syndrome (IBS) |
| Sanyal 2011 [[201](#_ENREF_201)]* | Gastroenterology | Nonalcoholic steatohepatitis |
| Rahn 2011 [[202](#_ENREF_202)]** | Gynaecology | Abnormal uterine bleeding |
| Meuleman 2012 [[203](#_ENREF_203)]** | Gynaecology | Deeply infiltrative endometriosis (DIE) |
| Vincent 2010 [[204](#_ENREF_204)]* | Gynaecology | Endometriosis-related pain |
| Broder 2000 [[205](#_ENREF_205)]* | Gynaecology | Uterine fibroids |
| Basson 2000 [[206](#_ENREF_206)]* | Gynaecology | Female sexual dysfunction |
| Clayton 2010 [[207](#_ENREF_207)]* | Gynaecology | Female sexual dysfunction |
| Walker 2006 [[208](#_ENREF_208)]** | Tobacco, drugs, & alcohol dependence | Addiction (gambling) |
| Del Boca 2007 [[209](#_ENREF_209)]* | Tobacco, drugs, & alcohol dependence | Addiction (substance) |
| Donovan 2012 [[210](#_ENREF_210)]** | Tobacco, drugs, & alcohol dependence | Drug dependence |
| Vocci 1999 [[211](#_ENREF_211)]* | Tobacco, drugs, & alcohol dependence | Nicotine, alcohol and cocaine abuse/dependence |
| Levine 2003 [[212](#_ENREF_212)]** | Urology | Peyronie's disease |
| Djurhuus 1997 [[213](#_ENREF_213)]** | Urology | Nocturnal enuresis |
| Toozs-Hobson 2012 [[214](#_ENREF_214)]** | Urology | Pelvic organ prolapse |
| Porst 2010 [[215](#_ENREF_215)]* | Urology | Male sexual dysfunction/disorders |
| Pavletic 2006 [[216](#_ENREF_216)]** | Blood disorders | Chronic graft-versus-host disease (GVHD) |
| Lassila 2005 [[217](#_ENREF_217)]* | Blood disorders | Haemophilia and other bleeding disorders |
| Rodeghiero 2009 [[218](#_ENREF_218)]** | Blood disorders | Immune thrombocytopenic purpura |
| Turk 2003 [[219](#_ENREF_219)]**  Turk 2008 [[220](#_ENREF_220)] | Anaesthesia & pain control | Chronic pain |
| McGrath 2008 [[221](#_ENREF_221)]** | Anaesthesia & pain control | Chronic pain/  recurrent pain and acute pain |
| Apfel 2002 [[222](#_ENREF_222)]* | Anaesthesia & pain control | Post-operative nausea and vomiting |
| Anderson 1998 [[223](#_ENREF_223)]* | Endocrine & metabolic | Obesity |
| Douglas 2009 [[224](#_ENREF_224)]** | Endocrine & metabolic | Thyroid eye disease (TED) |
| Carlson 2003 [[225](#_ENREF_225)]* | Mental health | Bipolar disorder |
| Rush 2006 [[226](#_ENREF_226)]** | Mental health | Major depressive disorder |
| Fitzpatrick 2010 [[227](#_ENREF_227)]** | Mental health | Forensic mental health |
| Finer 2006 [[228](#_ENREF_228)]*  Giacoia 2006 [[229](#_ENREF_229)] | Neonatal care | Neonatal apnea (also known as Apnea of prematurity, and Apnoea) |
| Short 2006 [[230](#_ENREF_230)]*  Giacoia 2006 | Neonatal care | Neonatal cardiovascular instability |
| Clancy 2006 [[231](#_ENREF_231)]*  Giacoia 2006 | Neonatal care | Neonatal seizures |
| Gonzalez 2011 [[232](#_ENREF_232)]  Eleftheriadou 2012 [[233](#_ENREF_233)]* | Skin | Vitiligo |
| Schmitt 2007 [[234](#_ENREF_234)]**  Schmitt 2010 [[235](#_ENREF_235)]  Schmitt 2011 [[236](#_ENREF_236)]  Schmitt 2012 [[237](#_ENREF_237)] | Skin | Eczema |
| Olliaro 2013 [[238](#_ENREF_238)]* | Skin | Cutaneous leishmaniasis |
| Bellomo 2004 [[239](#_ENREF_239)]* | Kidney disease | Acute renal failure |
| Molitoris 2012 [[240](#_ENREF_240)]* | Kidney disease | Acute kidney injury |
| Endre 2013 [[241](#_ENREF_241)]* | Kidney disease | Acute kidney injury |
| Abellan van Kan 2011 [[242](#_ENREF_242)]* | Health care of older people | Sarcopenia |
| Devane 2007 [[243](#_ENREF_243)]** | Pregnancy & child birth | Maternity care |
| Bennett 2012 [[244](#_ENREF_244)]* | Pregnancy & child birth | Gestational diabetes mellitus |
| Langguth 2007 [[245](#_ENREF_245)]** | Ear, nose & throat | Tinnitus |
| Ramsey 1994 [[246](#_ENREF_246)]** | Genetic disorders | Cystic Fibrosis |
| Gottrup 2010 [[247](#_ENREF_247)]** | Wounds | Non-healing wounds |
| van Brussel 2011 [[248](#_ENREF_248)]* | Chronic conditions | JIA, OI, Achondroplasia, Hemophilia, Cerebral, Palsy, Spina Bifida, CF, Cancer |
| Angus 2003 [[249](#_ENREF_249)]* | Intensive care | Critical illness/ ICU disease |
| Micke 2002 [[250](#_ENREF_250)]* | Benign disease | Benign/ Non-malignant diseases |

** Considered outcomes while addressing wider clinical trial design issues*

*** Specifically considered outcome selection and measurement*

**References**

1. Dixon DO, McLaughlin P, Hagemeister FB, Freireich EJ, Fuller LM, et al. (1987) Reporting outcomes in Hodgkin's disease and lymphoma. Journal of Clinical Oncology 5: 1670-1672.

2. Glynne-Jones R, Mawdsley S, Pearce T, Buyse M (2006) Alternative clinical end points in rectal cancer--are we getting closer? Annals of Oncology 17: 1239-1248.

3. Auvinen A, Rietbergen JB, Denis LJ, Schroder FH, Prorok PC (1996) Prospective evaluation plan for randomised trials of prostate cancer screening. The International Prostate Cancer Screening Trial Evaluation Group. Journal of Medical Screening 3: 97-104.

4. Denis L, Norlén BJ, Holmberg L, Begg CB, Damber JE, et al. (1997) Planning controlled clinical trials. Urology 49: 15-26.

5. Scher HI, Eisenberger M, D'Amico AV, Halabi S, Small EJ, et al. (2004) Eligibility and outcomes reporting guidelines for clinical trials for patients in the state of a rising prostate-specific antigen: recommendations from the Prostate-Specific Antigen Working Group.[Erratum appears in J Clin Oncol. 2004 Aug 1;22(15):3205]. Journal of Clinical Oncology 22: 537-556.

6. Dawson NA (1998) Apples and oranges: building a consensus for standardized eligibility criteria and end points in prostate cancer clinical trials. Journal of Clinical Oncology 16: 3398-3405.

7. Middleton RG, Thompson IM, Austenfeld MS, Cooner WH, Correa RJ, et al. (1995) Prostate Cancer Clinical Guidelines Panel Summary report on the management of clinically localized prostate cancer. The American Urological Association. Journal of Urology 154: 2144-2148.

8. Schellhammer P, Cockett A, Boccon-Gibod L, Gospodarowicz M, Krongrad A, et al. (1997) Assessment of endpoints for clinical trials for localized prostate cancer. Urology 49: 27-38.

9. Rajkumar SV, Harousseau J-L, Durie B, Anderson KC, Dimopoulos M, et al. (2011) Consensus recommendations for the uniform reporting of clinical trials: report of the International Myeloma Workshop Consensus Panel 1. Blood 117: 4691-4695.

10. Party IBMCW (2001) International bone metastases consensus on endpoint measurements for future clinical trials: proceedings of the first survey and meeting (work in progress) International Bone Metastases Consensus Working Party. Clinical Oncology (Royal College of Radiologists) 13: 82-84.

11. Chow E, Wu JSY, Hoskin P, Coia LR, Bentzen SM, et al. (2002) International consensus on palliative radiotherapy endpoints for future clinical trials in bone metastases. Radiotherapy & Oncology 64: 275-280.

12. Partsch H, Stout N, Forner-Cordero I, Flour M, Moffatt C, et al. (2010) Clinical trials needed to evaluate compression therapy in breast cancer related lymphedema (BCRL). Proposals from an expert group. International Angiology 29: 442-453.

13. Hesketh PJ, Gralla RJ, du Bois A, Tonato M (1998) Methodology of antiemetic trials: response assessment, evaluation of new agents and definition of chemotherapy emetogenicity. Supportive Care in Cancer 6: 221-227.

14. Tonato M (1998) Introduction. Supportive Care in Cancer 6: 193-193.

15. McVie JG, de Bruijn KM (1992) Methodology of antiemetic trials. Drugs 43 Suppl 3: 1-5.

16. Pallis AG, Ring A, Fortpied C, Penninckx B, Van Nes MC, et al. (2011) EORTC workshop on clinical trial methodology in older individuals with a diagnosis of solid tumors. Annals of Oncology 22: 1922-1926.

17. Miller AB, Hoogstraten B, Staquet M, Winkler A (1981) Reporting results of cancer treatment. Cancer 47: 207-214.

18. Prorok PC, Marcus PM (2010) Cancer screening trials: nuts and bolts. Seminars in Oncology 37: 216-223.

19. Punt CJA, Buyse M, Kohne C-H, Hohenberger P, Labianca R, et al. (2007) Endpoints in adjuvant treatment trials: a systematic review of the literature in colon cancer and proposed definitions for future trials. Journal of the National Cancer Institute 99: 998-1003.

20. Wils J, Sahmoud T, Sobrero A, Bleiberg H, Ahmedzai S, et al. (1998) Evaluation of clinical efficacy of new medical treatments in advanced colorectal cancer. Results of a workshop organized by the EORTC GITCCG. European Organization for Research and Treatment of Cancer. Gastrointestinal Tract Cancer Cooperative Group. Tumori 84: 335-347.

21. Llovet JM, Di Bisceglie AM, Bruix J, Kramer BS, Lencioni R, et al. (2008) Design and endpoints of clinical trials in hepatocellular carcinoma. Journal of the National Cancer Institute 100: 698-711.

22. Pagliusi SR, Teresa Aguado M (2004) Efficacy and other milestones for human papillomavirus vaccine introduction. Vaccine 23: 569-578.

23. Lefebvre J-L, Ang KK, Larynx Preservation Consensus P (2009) Larynx preservation clinical trial design: key issues and recommendations-a consensus panel summary. International Journal of Radiation Oncology, Biology, Physics 73: 1293-1303.

24. Adelstein DJ, Ridge JA, Brizel DM, Holsinger FC, Haughey BH, et al. (2012) Transoral resection of pharyngeal cancer: summary of a National Cancer Institute Head and Neck Cancer Steering Committee Clinical Trials Planning Meeting, November 6-7, 2011, Arlington, Virginia. Head & Neck 34: 1681-1703.

25. Gridelli C, Ardizzoni A, Le Chevalier T, Manegold C, Perrone F, et al. (2004) Treatment of advanced non-small-cell lung cancer patients with ECOG performance status 2: results of an European Experts Panel. Annals of Oncology 15: 419-426.

26. Gridelli C, de Marinis F, Di Maio M, Ardizzoni A, Belani CP, et al. (2012) Maintenance treatment of advanced non-small-cell lung cancer: Results of an International Expert Panel Meeting of the Italian Association of Thoracic Oncology. Lung Cancer 76: 269-279.

27. Cheson BD, Horning SJ, Coiffier B, Shipp MA, Fisher RI, et al. (1999) Report of an international workshop to standardize response criteria for non-Hodgkin's lymphomas. NCI Sponsored International Working Group. Journal of Clinical Oncology 17: 1244.

28. Cheson BD, Pfistner B, Juweid ME, Gascoyne RD, Specht L, et al. (2007) Revised response criteria for malignant lymphoma. Journal of Clinical Oncology 25: 579-586.

29. Anderson KC, Kyle RA, Rajkumar SV, Stewart AK, Weber D, et al. (2008) Clinically relevant end points and new drug approvals for myeloma. Leukemia 22: 231-239.

30. Cheson BD, Bennett JM, Kopecky KJ, Buchner T, Willman CL, et al. (2003) Revised recommendations of the International Working Group for Diagnosis, Standardization of Response Criteria, Treatment Outcomes, and Reporting Standards for Therapeutic Trials in Acute Myeloid Leukemia.[Erratum appears in J Clin Oncol. 2004 Feb 1;22(3):576 Note: LoCocco, Francesco [corrected to Lo-Coco, Francesco]]. Journal of Clinical Oncology 21: 4642-4649.

31. Kulke MH, Siu LL, Tepper JE, Fisher G, Jaffe D, et al. (2011) Future directions in the treatment of neuroendocrine tumors: consensus report of the National Cancer Institute Neuroendocrine Tumor clinical trials planning meeting. Journal of Clinical Oncology 29: 934-943.

32. Bellm LA, Cunningham G, Durnell L, Eilers J, Epstein JB, et al. (2002) Defining clinically meaningful outcomes in the evaluation of new treatments for oral mucositis: oral mucositis patient provider advisory board. Cancer Investigation 20: 793-800.

33. du Bois A, Quinn M, Thigpen T, Vermorken J, Avall-Lundqvist E, et al. (2005) 2004 consensus statements on the management of ovarian cancer: final document of the 3rd International Gynecologic Cancer Intergroup Ovarian Cancer Consensus Conference (GCIG OCCC 2004). Annals of Oncology 16 Suppl 8: viii7-viii12.

34. Stuart GCE, Kitchener H, Bacon M, duBois A, Friedlander M, et al. (2011) 2010 Gynecologic Cancer InterGroup (GCIG) consensus statement on clinical trials in ovarian cancer: report from the Fourth Ovarian Cancer Consensus Conference. International Journal of Gynecological Cancer 21: 750-755.

35. Thigpen T, duBois A, McAlpine J, DiSaia P, Fujiwara K, et al. (2011) First-line therapy in ovarian cancer trials. International Journal of Gynecological Cancer 21: 756-762.

36. Comenzo RL, Reece D, Palladini G, Seldin D, Sanchorawala V, et al. (2012) Consensus guidelines for the conduct and reporting of clinical trials in systemic light-chain amyloidosis. Leukemia 26: 2317-2325.

37. Dorman S, Jolley C, Abernethy A, Currow D, Johnson M, et al. (2009) Researching breathlessness in palliative care: consensus statement of the National Cancer Research Institute Palliative Care Breathlessness Subgroup. Palliative Medicine 23: 213-227.

38. Renal Disease Subcommittee of the American College of Rheumatology Ad Hoc Committee on Systemic Lupus Erythematosus Response C (2006) The American College of Rheumatology response criteria for proliferative and membranous renal disease in systemic lupus erythematosus clinical trials. Arthritis & Rheumatism 54: 421-432.

39. Bertsias GK, Ioannidis JPA, Boletis J, Bombardieri S, Cervera R, et al. (2009) EULAR points to consider for conducting clinical trials in systemic lupus erythematosus: literature based evidence for the selection of endpoints. Annals of the Rheumatic Diseases 68: 477-483.

40. Gordon C, Bertsias G, Ioannidis JPA, Boletis J, Bombardieri S, et al. (2009) EULAR points to consider for conducting clinical trials in systemic lupus erythematosus. Annals of the Rheumatic Diseases 68: 470-476.

41. Smolen JS, Strand V, Cardiel M, Edworthy S, Furst D, et al. (1999) Randomized clinical trials and longitudinal observational studies in systemic lupus erythematosus: consensus on a preliminary core set of outcome domains. Journal of Rheumatology 26: 504-507.

42. Ruperto N, Ravelli A, Murray KJ, Lovell DJ, Andersson-Gare B, et al. (2003) Preliminary core sets of measures for disease activity and damage assessment in juvenile systemic lupus erythematosus and juvenile dermatomyositis. Rheumatology 42: 1452-1459.

43. White B, Bauer EA, Goldsmith LA, Hochberg MC, Katz LM, et al. (1995) Guidelines for clinical trials in systemic sclerosis (scleroderma). I. Disease-modifying interventions. The American College of Rheumatology Committee on Design and Outcomes in Clinical Trials in Systemic Sclerosis. Arthritis & Rheumatism 38: 351-360.

44. Khanna D, Lovell DJ, Giannini E, Clements PJ, Merkel PA, et al. (2008) Development of a provisional core set of response measures for clinical trials of systemic sclerosis. Annals of the Rheumatic Diseases 67: 703-709.

45. Clements PJ, Allanore Y, Khanna D, Singh M, Furst DE (2012) Arthritis in Systemic Sclerosis: Systematic Review of the Literature and Suggestions for the Performance of Future Clinical Trials in Systemic Sclerosis Arthritis. Seminars in Arthritis and Rheumatism 41: 801-814.

46. Khanna D, Brown KK, Clements PJ, Elashoff R, Furst DE, et al. (2010) Systemic sclerosis-associated interstitial lung disease-proposed recommendations for future randomized clinical trials. Clinical & Experimental Rheumatology 28: S55-62.

47. Merkel PA, Herlyn K, Mahr AD, Neogi T, Seo P, et al. (2009) Progress towards a core set of outcome measures in small-vessel vasculitis. Report from OMERACT 9. Journal of Rheumatology 36: 2362-2368.

48. Merkel PA, Aydin SZ, Boers M, Direskeneli H, Herlyn K, et al. (2011) The OMERACT core set of outcome measures for use in clinical trials of ANCA-associated vasculitis. Journal of Rheumatology 38: 1480-1486.

49. Hellmich B, Flossmann O, Gross WL, Bacon P, Cohen-Tervaert JW, et al. (2007) EULAR recommendations for conducting clinical studies and/or clinical trials in systemic vasculitis: focus on anti-neutrophil cytoplasm antibody-associated vasculitis. Annals of the Rheumatic Diseases 66: 605-617.

50. Felson DT (1993) Choosing a core set of disease activity measures for rheumatoid arthritis clinical trials. Journal of Rheumatology 20: 531-534.

51. Fried BJ, Boers M, Baker PR (1993) A method for achieving consensus on rheumatoid arthritis outcome measures: the OMERACT conference process. Journal of Rheumatology 20: 548-551.

52. Tugwell P, Boers M (1993) Developing consensus on preliminary core efficacy endpoints for rheumatoid arthritis clinical trials. OMERACT Committee. Journal of Rheumatology 20: 555-556.

53. Boers M, Tugwell P, Felson DT, van Riel PL, Kirwan JR, et al. (1994) World Health Organization and International League of Associations for Rheumatology core endpoints for symptom modifying antirheumatic drugs in rheumatoid arthritis clinical trials. Journal of Rheumatology - Supplement 41: 86-89.

54. Kirwan J, Heiberg T, Hewlett S, Hughes R, Kvien T, et al. (2003) Outcomes from the Patient Perspective Workshop at OMERACT 6. Journal of Rheumatology 30: 868-872.

55. Kirwan JR, Hewlett SE, Heiberg T, Hughes RA, Carr M, et al. (2005) Incorporating the patient perspective into outcome assessment in rheumatoid arthritis - progress at OMERACT 7. Journal of Rheumatology. pp. 2250-2256.

56. Kirwan JR, Minnock P, Adebajo A, Bresnihan B, Choy E, et al. (2007) Patient perspective: fatigue as a recommended patient centered outcome measure in rheumatoid arthritis. Journal of Rheumatology 34: 1174-1177.

57. Sanderson T, Morris M, Calnan M, Richards P, Hewlett S (2010) Patient perspective of measuring treatment efficacy: the rheumatoid arthritis patient priorities for pharmacologic interventions outcomes. Arthritis care & research 62: 647-656.

58. Sanderson T, Morris M, Calnan M, Richards P, Hewlett S (2010) What outcomes from pharmacologic treatments are important to people with rheumatoid arthritis? Creating the basis of a patient core set. Arthritis care & research 62: 640-646.

59. Bombardier C, Tugwell P, Sinclair A, Dok C, Anderson G, et al. (1982) Preference for endpoint measures in clinical trials: results of structured workshops. Journal of Rheumatology 9: 798-801.

60. Scott DL, Spector TD, Pullar T, McConkey B (1989) What should we hope to achieve when treating rheumatoid arthritis? Annals of the Rheumatic Diseases 48: 256-261.

61. van Riel PL (1992) Provisional guidelines for measuring disease activity in clinical trials on rheumatoid arthritis. Br J Rheumatol 31: 793-794.

62. Taylor WJ (2005) Preliminary identification of core domains for outcome studies in psoriatic arthritis using Delphi methods. Annals of the Rheumatic Diseases 64 Suppl 2: ii110-112.

63. Gladman DD, Strand V, Mease PJ, Antoni C, Nash P, et al. (2005) OMERACT 7 psoriatic arthritis workshop: synopsis. Annals of the Rheumatic Diseases 64 Suppl 2: ii115-116.

64. Gladman DD (2005) Consensus exercise on domains in psoriatic arthritis. Annals of the Rheumatic Diseases 64 Suppl 2: ii113-114.

65. Gladman DD, Mease PJ, Strand V, Healy P, Helliwell PS, et al. (2007) Consensus on a core set of domains for psoriatic arthritis. Journal of Rheumatology 34: 1167-1170.

66. Miller FW, Rider LG, Chung YL, Cooper R, Danko K, et al. (2001) Proposed preliminary core set measures for disease outcome assessment in adult and juvenile idiopathic inflammatory myopathies. Rheumatology 40: 1262-1273.

67. Van Der Heijde D, Bellamy N, Calin A, Dougados M, Khan MA, et al. (1997) Preliminary core sets for endpoints in ankylosing spondylitis. Journal of Rheumatology 24: 2225-2229.

68. Wolfe F, Lassere M, van der Heijde D, Stucki G, Suarez-Almazor M, et al. (1999) Preliminary core set of domains and reporting requirements for longitudinal observational studies in rheumatology. Journal of Rheumatology 26: 484-489.

69. Mease PJ, Clauw DJ, Arnold LM, Goldenberg DL, Witter J, et al. (2005) Fibromyalgia syndrome. Journal of Rheumatology 32: 2270-2277.

70. Mease P, Arnold LM, Bennett R, Boonen A, Buskila D, et al. (2007) Fibromyalgia syndrome. Journal of Rheumatology 34: 1415-1425.

71. Arnold LM, Crofford LJ, Mease PJ, Burgess SM, Palmer SC, et al. (2008) Patient perspectives on the impact of fibromyalgia. Patient Education & Counseling 73: 114-120.

72. Mease PJ, Arnold LM, Crofford LJ, Williams DA, Russell IJ, et al. (2008) Identifying the clinical domains of fibromyalgia: contributions from clinician and patient Delphi exercises. Arthritis & Rheumatism 59: 952-960.

73. Carville SF, Choy E H (2008) Systematic review of discriminating power of outcome measures used in clinical trials of fibrymyalgia. Journal of Rheumatology 35: 2094-2105.

74. Choy EH, Arnold LM, Clauw DJ, Crofford LJ, Glass JM, et al. (2009) Content and criterion validity of the preliminary core dataset for clinical trials in fibromyalgia syndrome. Journal of Rheumatology 36: 2330-2334.

75. Mease P, Arnold LM, Choy EH, Clauw DJ, Crofford LJ, et al. (2009) Fibromyalgia syndrome module at OMERACT 9: domain construct. Journal of Rheumatology 36: 2318-2329.

76. Salaffi F, Ciapetti A, Sarzi Puttini P, Atzeni F, Iannuccelli C, et al. (2012) Preliminary identification of key clinical domains for outcome evaluation in fibromyalgia using the Delphi method: The Italian experience. Reumatismo 64: 27-34.

77. Schumacher HR, Edwards, N L, Perez-Ruiz, F et al (2005) Outcome measures for acute and chronic gout. Journal of Rheumatology 32: 2452-2455.

78. Schumacher HR, Taylor W, Joseph-Ridge N, Perez-Ruiz F, Chen LX, et al. (2007) Outcome evaluations in gout. Journal of Rheumatology 34: 1381-1385.

79. Taylor WJ, Schumacher HR, Jr., Baraf HSB, Chapman P, Stamp L, et al. (2008) A modified Delphi exercise to determine the extent of consensus with OMERACT outcome domains for studies of acute and chronic gout.[Erratum appears in Ann Rheum Dis. 2008 Nov;67(11):1652. Note: Mellado, J Vazquez [corrected to Vazquez-Mellado, J]]. Annals of the Rheumatic Diseases 67: 888-891.

80. Schumacher HR, Taylor W, Edwards L, Grainger R, Schlesinger N, et al. (2009) Outcome domains for studies of acute and chronic gout. Journal of Rheumatology 36: 2342-2345.

81. Giannini EH, Ruperto N, Ravelli A, Lovell DJ, Felson DT, et al. (1997) Preliminary definition of improvement in juvenile arthritis. Arthritis & Rheumatism 40: 1202-1209.

82. Bellamy N, Kirwan J, Boers M, Brooks P, Strand V, et al. (1997) Recommendations for a core set of outcome measures for future phase III clinical trials in knee, hip, and hand osteoarthritis. Consensus development at OMERACT III. Journal of Rheumatology 24: 799-802.

83. Heiligenhaus A, Foeldvari I, Edelsten C, Smith JR, Saurenmann RK, et al. (2012) Proposed outcome measures for prospective clinical trials in juvenile idiopathic arthritis-associated uveitis: a consensus effort from the multinational interdisciplinary working group for uveitis in childhood. Arthritis care & research 64: 1365-1372.

84. Bowman SJ, Pillemer S, Jonsson R, Asmussen K, Vitali C, et al. (2001) Revisiting Sjögren's syndrome in the new millennium: Perspectives on assessment and outcome measures. Report of a workshop held on 23 March 2000 at Oxford, UK. Rheumatology 40: 1180-1188.

85. Pillemer SR, Smith J, Fox PC, Bowman SJ (2005) Outcome measures for Sjogren's syndrome, April 10-11, 2003, Bethesda, Maryland, USA. Journal of Rheumatology 32: 143-149.

86. Cranney A, Tugwell P, Cummings S, Sambrook P, Adachi J, et al. (1997) Osteoporosis clinical trials endpoints: candidate variables and clinimetric properties. Journal of Rheumatology 24: 1222-1229.

87. (1997) Guidelines for osteoporosis trials. Journal of Rheumatology 24: 1234-1236.

88. Sjodahl Hammarlund C, Nilsson MH, Hagell P (2012) Measuring outcomes in Parkinson's disease: a multi-perspective concept mapping study. Quality of Life Research 21: 453-463.

89. Vellas B, Andrieu S, Sampaio C, Coley N, Wilcock G, et al. (2008) Endpoints for trials in Alzheimer's disease: a European task force consensus. Lancet Neurology 7: 436-450.

90. (1995) World Federation of Neurology Research Group on Neuromuscular Diseases Subcommittee on Motor Neuron Disease. Airlie House guidelines. Therapeutic trials in amyotrophic lateral sclerosis. Airlie House "Therapeutic Trials in ALS" Workshop Contributors. Journal of the Neurological Sciences 129 Suppl: 1-10.

91. Miller RG, Munsat TL, Swash M, Brooks BR (1999) Consensus guidelines for the design and implementation of clinical trials in ALS. World Federation of Neurology committee on Research. Journal of the Neurological Sciences 169: 2-12.

92. Leigh PN, Swash M, Iwasaki Y, Ludolph A, Meininger V, et al. (2004) Amyotrophic lateral sclerosis: a consensus viewpoint on designing and implementing a clinical trial. Amyotrophic Lateral Sclerosis & Other Motor Neuron Disorders 5: 84-98.

93. Shankaran S, Laptook A (2003) Challenge of conducting trials of neuroprotection in the asphyxiated term infant. Seminars in Perinatology 27: 320-332.

94. Vargus-Adams JN, Martin LK (2009) Measuring what matters in cerebral palsy: a breadth of important domains and outcome measures. Archives of Physical Medicine & Rehabilitation 90: 2089-2095.

95. Katona C, Livingston G, Cooper C, Ames D, Brodaty H, et al. (2007) International Psychogeriatric Association consensus statement on defining and measuring treatment benefits in dementia. International Psychogeriatrics 19: 345-354.

96. Moniz-Cook E, Vernooij-Dassen M, Woods R, Verhey F, Chattat R, et al. (2008) A European consensus on outcome measures for psychosocial intervention research in dementia care. Aging & Mental Health 12: 14-29.

97. (1998) Considerations on designing clinical trials to evaluate the place of new antiepileptic drugs in the treatment of newly diagnosed and chronic patients with epilepsy. Epilepsia 39: 799-803.

98. LaFrance WC, Jr., Alper K, Babcock D, Barry JJ, Benbadis S, et al. (2006) Nonepileptic seizures treatment workshop summary. Epilepsy & Behavior 8: 451-461.

99. Osborne JP, Lux A (2001) Towards an international consensus on definitions and standardised outcome measures for therapeutic trials (and epidemiological studies) in West syndrome. Brain & Development 23: 677-682.

100. Lux AL, Osborne JP (2004) A proposal for case definitions and outcome measures in studies of infantile spasms and West syndrome: consensus statement of the West Delphi group. Epilepsia 45: 1416-1428.

101. Mindell JA, Emslie G, Blumer J, Genel M, Glaze D, et al. (2006) Pharmacologic management of insomnia in children and adolescents: consensus statement. Pediatrics 117: e1223-1232.

102. Schumacher HC, Meyers PM, Higashida RT, Derdeyn CP, Lavine SD, et al. (2010) Reporting standards for angioplasty and stent-assisted angioplasty for intracranial atherosclerosis. Journal of Neurointerventional Surgery 2: 324-340.

103. Whitaker JN, McFarland HF, Rudge P, Reingold SC (1995) Outcomes assessment in multiple sclerosis clinical trials: a critical analysis. Multiple Sclerosis 1: 37-47.

104. Chitnis T, Tenembaum S, Banwell B, Krupp L, Pohl D, et al. (2012) Consensus statement: evaluation of new and existing therapeutics for pediatric multiple sclerosis. Multiple Sclerosis 18: 116-127.

105. Chitnis T, Tardieu M, Amato MP, Banwell B, Bar-Or A, et al. (2013) International Pediatric MS Study Group Clinical Trials Summit: meeting report. Neurology 80: 1161-1168.

106. Penzien DB (2005) Guidelines for trials of behavioral treatments for recurrent headache: purpose, process, and product. Headache 45 Suppl 2: S87-89.

107. Penzien DB, Andrasik F, Freidenberg BM, Houle TT, Lake AE, 3rd, et al. (2005) Guidelines for trials of behavioral treatments for recurrent headache, first edition: American Headache Society Behavioral Clinical Trials Workgroup. Headache 45 Suppl 2: S110-132.

108. Andrasik F, Lipchik GL, McCrory DC, Wittrock DA (2005) Outcome measurement in behavioral headache research: headache parameters and psychosocial outcomes. Headache 45: 429-437.

109. (1991) Guidelines for controlled trials of drugs in migraine. First edition. International Headache Society Committee on Clinical Trials in Migraine. Cephalalgia 11: 1-12.

110. Tfelt-Hansen P, Block G, Dahlof C, Diener HC, Ferrari MD, et al. (2000) Guidelines for controlled trials of drugs in migraine: second edition. Cephalalgia 20: 765-786.

111. Tfelt-Hansen P, Pascual J, Ramadan N, Dahlof C, D'Amico D, et al. (2012) Guidelines for controlled trials of drugs in migraine: third edition. A guide for investigators. Cephalalgia 32: 6-38.

112. Lipton RB, Micieli G, Russell D, Solomon S, Tfelt-Hansen P, et al. (1995) Guidelines for controlled trials of drugs in cluster headache. Cephalalgia 15: 452-462.

113. Schoenen J (1995) Guidelines for trials of drug treatments in tension-type headache. First edition: International Headache Society Committee on Clinical Trials. Cephalalgia 15: 165-179.

114. Bendtsen L, Bigal ME, Cerbo R, Diener HC, Holroyd K, et al. (2010) Guidelines for controlled trials of drugs in tension-type headache: second edition. Cephalalgia 30: 1-16.

115. Hughes R, Cidp, group MMNt (2005) 129th ENMC International Workshop: Clinical Trials for Chronic Inflammatory Demyelinating Polyradiculoneuropathy and Multifocal Motor Neuropathy, 27th October 2004, Schiphol airport, The Netherlands. Neuromuscular Disorders 15: 321-325.

116. Merkies ISJ, Lauria G (2006) 131st ENMC international workshop: selection of outcome measures for peripheral neuropathy clinical trials 10-12 December 2004, Naarden, The Netherlands. Neuromuscular Disorders 16: 149-156.

117. Reilly MM, de Jonghe P, Pareyson D (2006) 136th ENMC International Workshop: Charcot-Marie-Tooth disease type 1A (CMT1A)8-10 April 2005, Naarden, The Netherlands. Neuromuscular Disorders 16: 396-402.

118. Clifton GL, Hayes RL, Levin HS, Michel ME, Choi SC (1992) Outcome measures for clinical trials involving traumatically brain-injured patients: report of a conference. Neurosurgery 31: 975-978.

119. Wilde EA, Whiteneck GG, Bogner J, Bushnik T, Cifu DX, et al. (2010) Recommendations for the use of common outcome measures in traumatic brain injury research. Archives of Physical Medicine & Rehabilitation 91: 1650-1660 e1617.

120. Duncan PW, Jorgensen HS, Wade DT (2000) Outcome measures in acute stroke trials: a systematic review and some recommendations to improve practice. Stroke 31: 1429-1438.

121. Schellinger PD, Bath PMW, Lees KR, Bornstein NM, Uriel E, et al. (2012) Assessment of additional endpoints for trials in acute stroke - what, when, where, in who? International Journal of Stroke 7: 227-230.

122. Hoeper MM, Oudiz RJ, Peacock A, Tapson VF, Haworth SG, et al. (2004) End points and clinical trial designs in pulmonary arterial hypertension: clinical and regulatory perspectives. Journal of the American College of Cardiology 43: 48S-55S.

123. Distler O, Behrens F, Pittrow D, Huscher D, Denton CP, et al. (2008) Defining appropriate outcome measures in pulmonary arterial hypertension related to systemic sclerosis: a Delphi consensus study with cluster analysis.[Erratum appears in Arthritis Rheum. 2008 Aug 15;59(8):1202]. Arthritis & Rheumatism 59: 867-875.

124. Becker LB, Aufderheide TP, Geocadin RG, Callaway CW, Lazar RM, et al. (2011) Primary outcomes for resuscitation science studies: a consensus statement from the American Heart Association. Circulation 124: 2158-2177.

125. Chiam PTL, Ruiz CE (2008) Percutaneous transcatheter aortic valve implantation: assessing results, judging outcomes, and planning trials: the interventionalist perspective. Jacc: Cardiovascular Interventions 1: 341-350.

126. Leon MB, Piazza N, Nikolsky E, Blackstone EH, Cutlip DE, et al. (2011) Standardized endpoint definitions for transcatheter aortic valve implantation clinical trials: a consensus report from the Valve Academic Research Consortium. European Heart Journal 32: 205-217.

127. Kappetein AP, Head SJ, Genereux P, Piazza N, van Mieghem NM, et al. (2012) Updated standardized endpoint definitions for transcatheter aortic valve implantation: the Valve Academic Research Consortium-2 consensus document (VARC-2). European Journal of Cardio-Thoracic Surgery 42: S45-60.

128. Cutlip DE, Windecker S, Mehran R, Boam A, Cohen DJ, et al. (2007) Clinical end points in coronary stent trials: a case for standardized definitions. Circulation 115: 2344-2351.

129. Simons M, Bonow RO, Chronos NA, Cohen DJ, Giordano FJ, et al. (2000) Clinical trials in coronary angiogenesis: issues, problems, consensus: An expert panel summary. Circulation 102: E73-86.

130. Conte MS, Geraghty PJ, Bradbury AW, Hevelone ND, Lipsitz SR, et al. (2009) Suggested objective performance goals and clinical trial design for evaluating catheter-based treatment of critical limb ischemia. Journal of Vascular Surgery 50: 1462-1473.e1461-1463.

131. Timaran CH, McKinsey JF, Schneider PA, Littooy F (2011) Reporting standards for carotid interventions from the Society for Vascular Surgery. Journal of Vascular Surgery 53: 1679-1695.

132. Stout N, Partsch H, Szolnoky G, Forner-Cordero I, Mosti G, et al. (2012) Chronic edema of the lower extremities: international consensus recommendations for compression therapy clinical research trials. International Angiology 31: 316-329.

133. Nedeltchev K, Pattynama PM, Biaminoo G, Diehm N, Jaff MR, et al. (2010) Standardized definitions and clinical endpoints in carotid artery and supra-aortic trunk revascularization trials. Catheterization & Cardiovascular Interventions 76: 333-344.

134. Anderson HV, Weintraub WS, Radford MJ, Kremers MS, Roe MT, et al. (2013) Standardized cardiovascular data for clinical research, registries, and patient care: A report from the data standards workgroup of the national cardiovascular research infrastructure project. Journal of the American College of Cardiology 61: 1835-1846.

135. Hausenloy DJ, Erik Botker H, Condorelli G, Ferdinandy P, Garcia-Dorado D, et al. (2013) Translating cardioprotection for patient benefit: Position paper from the Working Group of Cellular Biology of the Heart of the European Society of Cardiology. Cardiovascular Research 98: 7-27.

136. Labs KH, Dormandy JA, Jaeger KA, Stuerzebecher C, Hiatt WR (1999) Trans-atlantic conference on clinical trial guidelines in PAOD (Peripheral arterial occlusive disease) clinical trial methodology. European Journal of Vascular & Endovascular Surgery 18: 253-265.

137. Mitchell LG, Goldenberg NA, Male C, Kenet G, Monagle P, et al. (2011) Definition of clinical efficacy and safety outcomes for clinical trials in deep venous thrombosis and pulmonary embolism in children. Journal of Thrombosis & Haemostasis 9: 1856-1858.

138. Steg PG, Huber K, Andreotti F, Arnesen H, Atar D, et al. (2011) Bleeding in acute coronary syndromes and percutaneous coronary interventions: position paper by the Working Group on Thrombosis of the European Society of Cardiology. European Heart Journal 32: 1854-1864.

139. O'Connell JB, McCarthy PM, Sopko G, Filippatos GS, Pina IL, et al. (2009) Mechanical circulatory support devices for acute heart failure syndromes: considerations for clinical trial design. Heart Failure Reviews 14: 101-112.

140. Higashida RT, Furlan AJ, Roberts H, Tomsick T, Connors B, et al. (2003) Trial design and reporting standards for intra-arterial cerebral thrombolysis for acute ischemic stroke.[Erratum appears in Stroke. 2003 Nov;34(11):2774]. Stroke 34: e109-137.

141. Calkins H, Kuck KH, Cappato R, Brugada J, Camm AJ, et al. (2012) 2012 HRS/EHRA/ECAS expert consensus statement on catheter and surgical ablation of atrial fibrillation: recommendations for patient selection, procedural techniques, patient management and follow-up, definitions, endpoints, and research trial design. Journal of Interventional Cardiac Electrophysiology 33: 171-257.

142. Kirchhof P, Auricchio A, Bax J, Crijns H, Camm J, et al. (2007) Outcome parameters for trials in atrial fibrillation: Executive summary - Recommendations from a consensus conference organized by the German Atrial Fibrillation Competence NETwork (AFNET) and the European Heart Rhythm Association (EHRA). European Heart Journal 28: 2803-2817.

143. Buser DA, Tonetti M (1997) Clinical trials on implants in regenerated bone. Annals of Periodontology 2: 329-342.

144. Page RC, DeRouen TA (1992) Design issues specific to studies of periodontitis. Journal of Periodontal Research 27: 395-404; discussion 412-396.

145. Imrey PB, Chilton NW, Pihlstrom BL, Proskin HM, Kingman A, et al. (1994) Proposed guidelines for American Dental Association acceptance of products for professional, non-surgical treatment of adult periodontitis. Task Force on Design and Analysis in Dental and Oral Research. Journal of Periodontal Research 29: 348-360.

146. Lightfoot WS, Hefti A, Mariotti A (2005) Using a Delphi panel to survey criteria for successful periodontal therapy in anterior teeth. Journal of Periodontology 76: 1508-1512.

147. Lightfoot WS, Hefti A, Mariotti A (2005) Using a Delphi panel to survey criteria for successful periodontal therapy in posterior teeth. Journal of Periodontology 76: 1502-1507.

148. Weber HP, Fiorellini JP, Jeffcoat MC (1997) Clinical trials on placement of implants in existing bone. Annals of Periodontology 2: 315-328.

149. Cochran DL (1998) Report of the American Academy of Periodontology's Workshop on the Design and Conduct of Clinical Trials for Endosseous Dental Implants. Journal of Periodontology 69: 280-284.

150. Tonetti M, Palmer R (2012) Clinical research in implant dentistry: Study design, reporting and outcome measurements: Consensus report of Working Group 2 of the VIII European Workshop on Periodontology. Journal of Clinical Periodontology 39: 73-80.

151. Smaïl-Faugeron V, Fron Chabouis H, Durieux P, Attal JP, Muller-Bolla M, et al. (2013) Development of a Core Set of Outcomes for Randomized Controlled Trials with Multiple Outcomes - Example of Pulp Treatments of Primary Teeth for Extensive Decay in Children. PLoS ONE 8.

152. Chilton NW, Fleiss JL (1986) Design and analysis of plaque and gingivitis clinical trials. J Clin Periodontol 13: 400-410.

153. (1986) Guidelines for acceptance of chemotherapeutic products for the control of supragingival dental plaque and gingivitis. Council on Dental Therapeutics. J Am Dent Assoc 112: 529-532.

154. Pitts NB, Stamm JW (2004) International Consensus Workshop on Caries Clinical Trials (ICW-CCT)--final consensus statements: agreeing where the evidence leads. J Dent Res 83 Spec No C: C125-128.

155. Marshall JC, Vincent J-L, Guyatt G, Angus DC, Abraham E, et al. (2005) Outcome measures for clinical research in sepsis: a report of the 2nd Cambridge Colloquium of the International Sepsis Forum. Critical Care Medicine 33: 1708-1716.

156. Goldstein B, Giroir B, Randolph A, International Consensus Conference on Pediatric S (2005) International pediatric sepsis consensus conference: definitions for sepsis and organ dysfunction in pediatrics. Pediatric Critical Care Medicine 6: 2-8.

157. Wood MJ, Balfour H, Beutner K, Bruxelle J, Fiddian P, et al. (1995) How should zoster trials be conducted? Journal of Antimicrobial Chemotherapy 36: 1089-1101.

158. Barlow GD, Lamping DL, Davey PG, Nathwani D (2003) Evaluation of outcomes in community-acquired pneumonia: a guide for patients, physicians, and policy-makers. The Lancet Infectious Diseases 3: 476-488.

159. Spellberg B, Talbot GH, Brass EP, Bradley JS, Boucher HW, et al. (2008) Position paper: recommended design features of future clinical trials of antibacterial agents for community-acquired pneumonia. Clinical Infectious Diseases 47 Suppl 3: S249-265.

160. Powers JH (2010) Recommendations for improving the design, conduct, and analysis of clinical trials in hospital-acquired pneumonia and ventilator-associated pneumonia. Clinical Infectious Diseases 51 Suppl 1: S18-28.

161. Spellberg B, Talbot G, Infectious Diseases Society of A, American College of Chest P, American Thoracic S, et al. (2010) Recommended design features of future clinical trials of antibacterial agents for hospital-acquired bacterial pneumonia and ventilator-associated bacterial pneumonia. Clinical Infectious Diseases 51 Suppl 1: S150-170.

162. McCracken GH, Sande MA, Lentnek A, Whitley RJ, Scheld WM (1992) Evaluation of new anti-infective drugs for the treatment of acute bacterial meningitis. Infectious Diseases Society of America and the Food and Drug Administration. Clinical Infectious Diseases 15 Suppl 1: S182-188.

163. Alioum A, Dabis F, Dequae-Merchadou L, Haverkamp G, Hudgens M, et al. (2001) Estimating the efficacy of interventions to prevent mother-to-child transmission of HIV in breast-feeding population: Development of a consensus methodology. Statistics in Medicine 20: 3539-3556.

164. Kirkby R, Calabrese C, Kaltman L, Monnier J, Herscu P (2010) Methodological considerations for future controlled influenza treatment trials in complementary and alternative medicine. Journal of Alternative & Complementary Medicine 16: 275-283.

165. Nystrom PO, Bax R, Dellinger EP, Dominioni L, Knaus WA, et al. (1990) Proposed definitions for diagnosis, severity scoring, stratification, and outcome for trials on intraabdominal infection. Joint Working Party of SIS North America and Europe. World Journal of Surgery 14: 148-158.

166. Cross H (2005) A Delphi consensus on criteria for contraindications, assessment indicators and expected outcomes related to tibialis posterior transfer surgery. International Journal of Leprosy & Other Mycobacterial Diseases 73: 13-21.

167. Moorthy V, Reed Z, Smith PG, Efficacy WHOSGoMoMV (2007) Measurement of malaria vaccine efficacy in phase III trials: report of a WHO consultation. Vaccine 25: 5115-5123.

168. Moorthy VS, Reed Z, Smith PG, Committee WHOMVA (2009) MALVAC 2008: Measures of efficacy of malaria vaccines in phase 2b and phase 3 trials--scientific, regulatory and public health perspectives. Vaccine 27: 624-628.

169. Steeves JD, Lammertse D, Curt A, Fawcett JW, Tuszynski MH, et al. (2007) Guidelines for the conduct of clinical trials for spinal cord injury (SCI) as developed by the ICCP panel: clinical trial outcome measures. Spinal Cord 45: 206-221.

170. Bombardier C (2000) Outcome assessments in the evaluation of treatment of spinal disorders: summary and general recommendations. Spine 25: 3100-3103.

171. Deyo RA, Battie M, Beurskens AJ, Bombardier C, Croft P, et al. (1998) Outcome measures for low back pain research. A proposal for standardized use. Spine 23: 2003-2013.

172. Devogelaer JP, Dreiser RL, Abadie E, Avouac B, Bouvenot G, et al. (2003) Guidelines for clinical studies assessing the efficacy of drugs for the management of acute low back pain. Clinical & Experimental Rheumatology 21: 691-694.

173. Goldhahn J, Scheele WH, Mitlak BH, Abadie E, Aspenberg P, et al. (2008) Clinical evaluation of medicinal products for acceleration of fracture healing in patients with osteoporosis. Bone 43: 343-347.

174. Lynch AD, Logerstedt DS, Grindem H, Eitzen I, Hicks GE, et al. (2013) Consensus criteria for defining 'successful outcome' after ACL injury and reconstruction: A Delaware-Oslo ACL cohort investigation. British Journal of Sports Medicine.

175. Falder S, Browne A, Edgar D, Staples E, Fong J, et al. (2009) Core outcomes for adult burn survivors: a clinical overview. Burns 35: 618-641.

176. Reneman MF, Beemster TT, Edelaar MJA, van Velzen JM, van Bennekom C, et al. (2013) Towards an ICF- and IMMPACT-Based Pain Vocational Rehabilitation Core Set in the Netherlands. Journal of Occupational Rehabilitation: 1-9.

177. Lamb SE, Jorstad-Stein EC, Hauer K, Becker C, Prevention of Falls Network E, et al. (2005) Development of a common outcome data set for fall injury prevention trials: the Prevention of Falls Network Europe consensus. Journal of the American Geriatrics Society 53: 1618-1622.

178. Cameron ID, Robinovitch S, Birge S, Kannus P, Khan K, et al. (2010) Hip protectors: recommendations for conducting clinical trials--an international consensus statement (part II). Osteoporosis International 21: 1-10.

179. Smith MA, Leeder SR, Jalaludin B, Smith WT (1996) The asthma health outcome indicators study. Australian & New Zealand Journal of Public Health 20: 69-75.

180. Reddel HK, Taylor DR, Bateman ED, Boulet LP, Boushey HA, et al. (2009) An official American Thoracic Society/European Respiratory Society statement: asthma control and exacerbations: standardizing endpoints for clinical asthma trials and clinical practice. Am J Respir Crit Care Med 180: 59-99.

181. Busse WW, Morgan WJ, Taggart V, Togias A (2012) Asthma outcomes workshop: overview. Journal of Allergy & Clinical Immunology 129: S1-8.

182. Fuhlbrigge A, Peden D, Apter AJ, Boushey HA, Camargo CA, Jr., et al. (2012) Asthma outcomes: exacerbations. Journal of Allergy & Clinical Immunology 129: S34-48.

183. Akinbami LJ, Sullivan SD, Campbell JD, Grundmeier RW, Hartert TV, et al. (2012) Asthma outcomes: healthcare utilization and costs. Journal of Allergy & Clinical Immunology 129: S49-64.

184. Szefler SJ, Wenzel S, Brown R, Erzurum SC, Fahy JV, et al. (2012) Asthma outcomes: biomarkers. Journal of Allergy & Clinical Immunology 129: S9-23.

185. Tepper RS, Wise RS, Covar R, Irvin CG, Kercsmar CM, et al. (2012) Asthma outcomes: pulmonary physiology. Journal of Allergy & Clinical Immunology 129: S65-87.

186. Cloutier MM, Schatz M, Castro M, Clark N, Kelly HW, et al. (2012) Asthma outcomes: Composite scores of asthma control. Journal of Allergy and Clinical Immunology 129: S24-S33.

187. Krishnan JA, Lemanske Jr RF, Canino GJ, Elward KS, Kattan M, et al. (2012) Asthma outcomes: Symptoms. Journal of Allergy and Clinical Immunology 129: S124-S135.

188. Wilson SR, Rand CS, Cabana MD, Foggs MB, Halterman JS, et al. (2012) Asthma outcomes: Quality of life. Journal of Allergy and Clinical Immunology 129: S88-S123.

189. Sinha IP, Gallagher R, Williamson PR, Smyth RL (2012) Development of a core outcome set for clinical trials in childhood asthma: a survey of clinicians, parents, and young people. Trials 13: 103.

190. Keim SM, Spaite DW, Maio RF, Garrison HG, Desmond JS, et al. (2004) Risk adjustment and outcome measures for out-of-hospital respiratory distress. Academic Emergency Medicine 11: 1074-1081.

191. Canonica GW, Baena-Cagnani CE, Bousquet J, Bousquet PJ, Lockey RF, et al. (2007) Recommendations for standardization of clinical trials with Allergen Specific Immunotherapy for respiratory allergy. A statement of a World Allergy Organization (WAO) taskforce. Allergy 62: 317-324.

192. Cazzola M, MacNee W, Martinez FJ, Rabe KF, Franciosi LG, et al. (2008) Outcomes for COPD pharmacological trials: from lung function to biomarkers. European Respiratory Journal 31: 416-469.

193. (1994) Recommendations for guidelines on clinical trials of mucoactive drugs in chronic bronchitis and chronic obstructive pulmonary disease. Task Group on Mucoactive Drugs. Chest 106: 1532-1537.

194. Dent J, Kahrilas PJ, Vakil N, Van Zanten SV, Bytzer P, et al. (2008) Clinical trial design in adult reflux disease: a methodological workshop. Alimentary Pharmacology & Therapeutics 28: 107-126.

195. Wirth S, Kelly D, Sokal E, Socha P, Mieli-Vergani G, et al. (2011) Guidance for clinical trials for children and adolescents with chronic hepatitis C. Journal of Pediatric Gastroenterology & Nutrition 52: 233-237.

196. Bajaj JS, Cordoba J, Mullen KD, Amodio P, Shawcross DL, et al. (2011) Review article: the design of clinical trials in hepatic encephalopathy--an International Society for Hepatic Encephalopathy and Nitrogen Metabolism (ISHEN) consensus statement. Alimentary Pharmacology & Therapeutics 33: 739-747.

197. Fekety R, DuPont HL, Cooperstock M, Corrado ML, Murray DM (1992) Evaluation of new anti-infective drugs for the treatment of antibiotic-associated colitis. Infectious Diseases Society of America and the Food and Drug Administration. Clinical Infectious Diseases 15 Suppl 1: S263-267.

198. Griffiths AM, Otley AR, Hyams J, Quiros AR, Grand RJ, et al. (2005) A review of activity indices and end points for clinical trials in children with Crohn's disease. Inflammatory Bowel Diseases 11: 185-196.

199. Laine L, Spiegel B, Rostom A, Moayyedi P, Kuipers EJ, et al. (2010) Methodology for randomized trials of patients with nonvariceal upper gastrointestinal bleeding: recommendations from an international consensus conference. American Journal of Gastroenterology 105: 540-550.

200. Pimentel M, Talley NJ, Quigley EM, Hani A, Sharara A, et al. (2013) Report from the multinational irritable bowel syndrome initiative 2012. Gastroenterology 144: e1-5.

201. Sanyal AJ, Brunt EM, Kleiner DE, Kowdley KV, Chalasani N, et al. (2011) Endpoints and clinical trial design for nonalcoholic steatohepatitis. Hepatology 54: 344-353.

202. Rahn DD, Abed H, Sung VW, Matteson KA, Rogers RG, et al. (2011) Systematic review highlights difficulty interpreting diverse clinical outcomes in abnormal uterine bleeding trials. Journal of Clinical Epidemiology 64: 293-300.

203. Meuleman C, Tomassetti C, D'Hooghe TM (2012) Clinical outcome after laparoscopic radical excision of endometriosis and laparoscopic segmental bowel resection. Current Opinion in Obstetrics & Gynecology 24: 245-252.

204. Vincent K, Kennedy S, Stratton P (2010) Pain scoring in endometriosis: entry criteria and outcome measures for clinical trials. Report from the Art and Science of Endometriosis meeting. Fertility & Sterility 93: 62-67.

205. Broder MS, Landow WJ, Goodwin SC, Brook RH, Sherbourne CD, et al. (2000) An agenda for research into uterine artery embolization: results of an expert panel conference. Journal of Vascular & Interventional Radiology 11: 509-515.

206. Basson R, Berman J, Burnett A, Derogatis L, Ferguson D, et al. (2000) Report of the international consensus development conference on female sexual dysfunction: definitions and classifications. Journal of Urology 163: 888-893.

207. Clayton AH, Dennerstein L, Fisher WA, Kingsberg SA, Perelman MA, et al. (2010) Standards for clinical trials in sexual dysfunction in women: research designs and outcomes assessment. Journal of Sexual Medicine 7: 541-560.

208. Walker M, Toneatto T, Potenza MN, Petry N, Ladouceur R, et al. (2006) A framework for reporting outcomes in problem gambling treatment research: the Banff, Alberta Consensus. Addiction 101: 504-511.

209. Del Boca FK, Darkes J (2007) Enhancing the validity and utility of randomized clinical trials in addictions treatment research: II. Participant samples and assessment. Addiction 102: 1194-1203.

210. Donovan DM, Bigelow GE, Brigham GS, Carroll KM, Cohen AJ, et al. (2012) Primary outcome indices in illicit drug dependence treatment research: systematic approach to selection and measurement of drug use end-points in clinical trials. Addiction 107: 694-708.

211. Vocci F, Wit Hd (1999) Consensus statement on evaluation of outcome of pharmacotherapy for substance abuse/ dependence: report from a NIDA/CPDD meeting. National Institute on Drug Abuse Medications Development Division.

212. Levine LA, Greenfield JM (2003) Establishing a standardized evaluation of the man with Peyronie's disease. International Journal of Impotence Research 15 Suppl 5: S103-112.

213. Djurhuus JC, Norgaard JP, Hjalmas K (1997) What is an acceptable treatment outcome? Scandinavian Journal of Urology & Nephrology Supplementum 183: 75-77.

214. Toozs-Hobson P, Freeman R, Barber M, Maher C, Haylen B, et al. (2012) An International Urogynecological Association (IUGA)/International Continence Society (ICS) joint report on the terminology for reporting outcomes of surgical procedures for pelvic organ prolapse. International Urogynecology Journal 23: 527-535.

215. Porst H, Vardi Y, Akkus E, Melman A, Park NC, et al. (2010) Standards for clinical trials in male sexual dysfunctions. Journal of Sexual Medicine 7: 414-444.

216. Pavletic SZ, Martin P, Lee SJ, Mitchell S, Jacobsohn D, et al. (2006) Measuring therapeutic response in chronic graft-versus-host disease: National Institutes of Health Consensus Development Project on Criteria for Clinical Trials in Chronic Graft-versus-Host Disease: IV. Response Criteria Working Group report. Biology of Blood & Marrow Transplantation 12: 252-266.

217. Lassila R, Rothschild C, De Moerloose P, Richards M, Perez R, et al. (2005) Recommendations for postmarketing surveillance studies in haemophilia and other bleeding disorders. Haemophilia 11: 353-359.

218. Rodeghiero F, Stasi R, Gernsheimer T, Michel M, Provan D, et al. (2009) Standardization of terminology, definitions and outcome criteria in immune thrombocytopenic purpura of adults and children: report from an international working group. Blood 113: 2386-2393.

219. Turk DC, Dworkin RH, Allen RR, Bellamy N, Brandenburg N, et al. (2003) Core outcome domains for chronic pain clinical trials: IMMPACT recommendations. Pain 106: 337-345.

220. Turk DC, Dworkin RH, Revicki D, Harding G, Burke LB, et al. (2008) Identifying important outcome domains for chronic pain clinical trials: an IMMPACT survey of people with pain. Pain 137: 276-285.

221. McGrath PJ, Walco GA, Turk DC, Dworkin RH, Brown MT, et al. (2008) Core outcome domains and measures for pediatric acute and chronic/recurrent pain clinical trials: PedIMMPACT recommendations. Journal of Pain 9: 771-783.

222. Apfel C, Eberhart L, Kranke P, Ruesch D (2002) Recommendations for randomized controlled trials to prevent or treat postoperative nausea and vomiting. Anaesth Intensivmed Notfallmed Schmerzther 43: 69-74.

223. Anderson JW, Pi-Sunyer FX, Danforth E, Dujovne CA, Greenway F, et al. (1998) Clinical trial design for obesity agents: a workshop report. Obesity Research 6: 311-315.

224. Douglas RS, Tsirbas A, Gordon M, Lee D, Khadavi N, et al. (2009) Development of criteria for evaluating clinical response in thyroid eye disease using a modified Delphi technique. Archives of Ophthalmology 127: 1155-1160.

225. Carlson GA, Jensen PS, Findling RL, Meyer RE, Calabrese J, et al. (2003) Methodological issues and controversies in clinical trials with child and adolescent patients with bipolar disorder: report of a consensus conference. Journal of Child & Adolescent Psychopharmacology 13: 13-27.

226. Rush AJ, Kraemer HC, Sackeim HA, Fava M, Trivedi MH, et al. (2006) Report by the ACNP Task Force on response and remission in major depressive disorder. Neuropsychopharmacology 31: 1841-1853.

227. Fitzpatrick R, Chambers J, Burns T, Doll H, Fazel S, et al. (2010) A systematic review of outcome measures used in forensic mental health research with consensus panel opinion. Health Technology Assessment (Winchester, England) 14: 1-94.

228. Finer NN, Higgins R, Kattwinkel J, Martin RJ (2006) Summary proceedings from the apnea-of-prematurity group. Pediatrics 117: S47-51.

229. Giacoia GP, Birenbaum DL, Sachs HC, Mattison DR (2006) The newborn drug development initiative. Pediatrics 117: S1-8.

230. Short BL, Van Meurs K, Evans JR, Cardiology G (2006) Summary proceedings from the cardiology group on cardiovascular instability in preterm infants. Pediatrics 117: S34-39.

231. Clancy RR (2006) Summary proceedings from the neurology group on neonatal seizures. Pediatrics 117: S23-S27.

232. Gonzalez U, Whitton M, Eleftheriadou V, Pinart M, Batchelor J, et al. (2011) Guidelines for designing and reporting clinical trials in vitiligo. Archives of Dermatology 147: 1428-1436.

233. Eleftheriadou V, Thomas KS, Whitton ME, Batchelor JM, Ravenscroft JC (2012) Which outcomes should we measure in vitiligo? Results of a systematic review and a survey among patients and clinicians on outcomes in vitiligo trials. British Journal of Dermatology 167: 804-814.

234. Schmitt J, Langan S, Williams HC, European Dermato-Epidemiology N (2007) What are the best outcome measurements for atopic eczema? A systematic review. Journal of Allergy & Clinical Immunology 120: 1389-1398.

235. Schmitt J, Williams H, Group HD (2010) Harmonising Outcome Measures for Eczema (HOME). Report from the First International Consensus Meeting (HOME 1), 24 July 2010, Munich, Germany. British Journal of Dermatology 163: 1166-1168.

236. Schmitt J, Langan S, Stamm T, Williams HC, Harmonizing Outcome Measurements in Eczema Delphi p (2011) Core outcome domains for controlled trials and clinical recordkeeping in eczema: international multiperspective Delphi consensus process. Journal of Investigative Dermatology 131: 623-630.

237. Schmitt J, Spuls P, Boers M, Thomas K, Chalmers J, et al. (2012) Towards global consensus on outcome measures for atopic eczema research: results of the HOME II meeting. Allergy 67: 1111-1117.

238. Olliaro P, Vaillant M, Arana B, Grogl M, Modabber F, et al. (2013) Methodology of clinical trials aimed at assessing interventions for cutaneous leishmaniasis. PLoS Neglected Tropical Diseases [electronic resource] 7: e2130.

239. Bellomo R, Ronco C, Kellum JA, Mehta RL, Palevsky P, et al. (2004) Acute renal failure - definition, outcome measures, animal models, fluid therapy and information technology needs: the Second International Consensus Conference of the Acute Dialysis Quality Initiative (ADQI) Group. Critical Care (London, England) 8: R204-212.

240. Molitoris BA, Okusa MD, Palevsky PM, Kimmel PL, Star RA (2012) Designing clinical trials in acute kidney injury. Clinical Journal of the American Society of Nephrology 7: 842-843.

241. Endre ZH, Pickering JW (2013) Acute kidney injury clinical trial design: old problems, new strategies. Pediatric Nephrology 28: 207-217.

242. Abellan van Kan G, Cameron Chumlea W, Gillette-Guyonet S, Houles M, Dupuy C, et al. (2011) Clinical trials on sarcopenia: methodological issues regarding phase 3 trials. Clinics in Geriatric Medicine 27: 471-482.

243. Devane D, Begley CM, Clarke M, Horey D, Oboyle C (2007) Evaluating maternity care: a core set of outcome measures. Birth 34: 164-172.

244. Bennett WL, Robinson KA, Saldanha IJ, Wilson LM, Nicholson WK (2012) High priority research needs for gestational diabetes mellitus. Journal of Women's Health 21: 925-932.

245. Langguth B, Goodey R, Azevedo A, Bjorne A, Cacace A, et al. (2007) Consensus for tinnitus patient assessment and treatment outcome measurement: Tinnitus Research Initiative meeting, Regensburg, July 2006. Progress in Brain Research 166: 525-536.

246. Ramsey BW, Boat TF (1994) Outcome measures for clinical trials in cystic fibrosis. Summary of a Cystic Fibrosis Foundation consensus conference. Journal of Pediatrics 124: 177-192.

247. Gottrup F, Apelqvist J, Price P, European Wound Management Association Patient Outcome G (2010) Outcomes in controlled and comparative studies on non-healing wounds: recommendations to improve the quality of evidence in wound management. Journal of Wound Care 19: 237-268.

248. van Brussel M, van der Net J, Hulzebos E, Helders PJM, Takken T (2011) The Utrecht approach to exercise in chronic childhood conditions: the decade in review. Pediatric Physical Therapy 23: 2-14.

249. Angus DC, Carlet J, Brussels Roundtable P (2003) Surviving intensive care: a report from the 2002 Brussels Roundtable. Intensive Care Medicine 29: 368-377.

250. Micke O, Seegenschmiedt MH, German Working Group on Radiotherapy in G (2002) Consensus guidelines for radiation therapy of benign diseases: a multicenter approach in Germany. International Journal of Radiation Oncology, Biology, Physics 52: 496-513.
